# Hydrophobic residues are critical for the helix-forming, hemolytic and bactericidal activities of amphipathic antimicrobial peptide TP4

**DOI:** 10.1371/journal.pone.0186442

**Published:** 2017-10-17

**Authors:** Ting-Wei Chang, Shu-Yi Wei, Shih-Han Wang, Hung-Mu Wei, Yu-June Wang, Chiu-Feng Wang, Chinpan Chen, You-Di Liao

**Affiliations:** Institute of Biomedical Sciences, Academia Sinica, Taipei, Taiwan; George Washington University, UNITED STATES

## Abstract

Antimicrobial peptides are important components of the host innate defense mechanism against invading pathogens, especially for drug-resistant bacteria. In addition to bactericidal activity, the 25 residue peptide TP4 isolated from Nile tilapia also stimulates cell proliferation and regulates the innate immune system in mice. In this report, TP4 hyperpolarized and depolarized the membrane potential of *Pseudomonas aeruginosa* at sub-lethal and lethal concentrations. It also inhibited and eradicated biofilm formation. The *in vitro* binding of TP4 to bacterial outer membrane target protein, OprI, was markedly enhanced by a membrane-like surfactant sarkosyl and lipopolysaccharide, which converted TP4 into an α-helix. The solution structure of TP4 in dodecylphosphocholine was solved by NMR analyses. It contained a typical α-helix at residues Phe10-Arg22 and a distorted helical segment at Ile6-Phe10, as well as a hydrophobic core at the N-terminus and a cationic patch at the C-terminus. Residues Ile16, Leu19 and Ile20 in the hydrophobic face of the main helix were critical for the integrity of amphipathic structure, other hydrophobic residues played important roles in hemolytic and bactericidal activities. A model for the assembly of helical TP4 embedded in sarkosyl vesicle is proposed. This study may provide valuable insight for engineering AMPs to have potent bactericidal activity but low hemolytic activity.

## Introduction

Conventional antibiotics can inhibit the synthesis of bacterial nucleic acids, proteins, or cell wall components. However, the widespread use of antibiotics in both medicine and agriculture have caused the emergence of drug-resistant bacteria [1,2]. Thus, development of new antimicrobials with unique targets and mechanisms of action is an acute and urgent need. Naturally occurring antimicrobial peptides/proteins (AMPs) can disrupt the bacterial membrane integrity and may fill this need. It has been isolated from multiple sources including bacteria, fungi, insects, invertebrates and vertebrates with remarkably diverse structures and bioactivity profiles [2–4]. These bioactive peptides do not merely act as direct antimicrobial agents but also represent important effectors and regulators of the innate immune system, similar to LL37 [5,6].

The piscidin family of AMPs from fish consists of structurally related peptides of 21~44 residues that possess amphipathic structures. Five different piscidins, named TP1~5, are isolated from the Nile tilapia *Oreochromis niloticus*. The TP4 among them exerts the strongest antimicrobial activity against both Gram-positive and -negative bacteria as well as hemolytic activity against fish red blood cells and cytotoxicity towards fish ovary cells [7]. In addition, it also stimulates cell proliferation and wound closure in MRSA-infected wounds in mice [8]. During infection of *Helicobacter pylori*, TP4 is able to suppress immune response and clear bacteria in mouse models [9]. Although TP4 possesses great potential in the treatment of bacterial infection, it also exerts high hemolytic and cytotoxic effects. Therefore, it is crucial to define the residues responsible for the above-mentioned activities and modify the peptides to generate increasing bactericidal ability, but with decreased hemolytic and cytotoxic activities.

We report here the membrane potential of *Pseudomonas aeruginosa* to be hyperpolarized or depolarized by TP4 depending on the concentration employed. The *in vitro* binding of TP4 to the bacterial outer membrane target protein, OprI, was enhanced by a membrane-like surfactant sarkosyl and lipopolysaccharide (LPS), which could drive TP4 into an α-helical structure. By NMR analyses, we found TP4 to contain a typical α-helical structure with residues Phe10-Arg22 and a distorted helical segment with residues at Ile6-Phe10. Residues responsible for the integrity of amphipathic structure as well as hemolytic and bactericidal activities were determined.

## Materials and methods

### Ethics statement

Whole blood was collected from each of 20 wild type C57BL/6JNarl mice obtained from National Laboratory Animal Center. The experimental procedures were conducted by National Institutes of Health guidelines, in accordance with the guidelines specified by the Institutional Animal Care and Utilization Committee, Academia Sinica (Taipei, Taiwan) with the permit number IACUC 13-12-628.

### Materials

TP4 (FIHHIIGGLFSAGKAIHRLIRRRRR) and its derived peptides were synthesized by Kelowna International Scientific Inc. (Taipei, Taiwan) with more than 95% purity and their molecular weights were verified by mass spectrum analysis. Sodium dodecyl sulfate (SDS) was obtained from Merck (Darmstadt, Germany). Streptavidin gel was purchased from GE Healthcare (Uppsala, Sweden). Sodium N-dodecanoylsarcosinate (sarkosyl) was supplied by Wako Pure Chem. (Osaka, Japan). 1-ethyl-3-[3-dimethylaminopropyl] carbodiimide hydrochloride (EDC) was purchased from Thermo Fisher (St. Waltham, MA, USA). 3,3’-dipropylthiadicarbocyanine iodide (DiSC3(5)) was obtained from Molecular Probes (OR, USA). Dodecylphosphocholine (DPC) was purchased from Avanti Polar Lipids, Inc. Crystal violet, MnCl_2_ and 8-anilino-1-naphthalenesulfonic acid (ANS) were purchased from Sigma-Aldrich Inc. Dodecylphosphocholine-d38 (DPC) and D_2_O were supplied by Cambridge Isotope Laboratories, Inc.

### Antimicrobial activity assays

Bacteria were cultured in Luria-Bertani broth and plated on Luria-Bertani agar for *Pseudomonas aeruginosa* PAO1 (ATCC BAA-47TM), *P*. *aeruginosa* (ATCC 27853), *Klebsiell*a *pneumoniae* (ATCC 13884) and *Staphylococcus aureus* (ATCC49476). methicillin-resistant *Staphylococcus aureus* (MRSA), vancomycin-resistant enterococcus clinical isolate VRE 2061007 from National Taiwan University Hospital was cultured and plated in/on tryptic soy broth/agar (Difco0369). *Listeria monocytogenes* was cultured in/on Bacto^TM^ BHI broth/agar (BD). *Candida albicans* were cultured in/on Difco ^TM^ YM broth/agar. The microbes were grown overnight, washed and diluted 300 folds in 10 mM sodium phosphate, pH 7.4 with 45 μl of the microbes (5–10 x10^4^ colony forming units (cfu)) mixed with serially diluted TP4 (5 μl) and incubated at 30/37°C for 1.5 hr. Serial dilutions of each AMP-treated bacteria were prepared and plated on agar plates for the determination of remaining cfu. At least three independent experiments were performed for each assay to determine the average value with standard deviation [10]. Alternatively, the bactericidal activity of TP4 against higher concentration of *P*. *aeruginosa* PAO1 (5 x10^6^ cfu/45μl) was performed in the membrane potential assay buffer (5 mM Hepes, pH 7.2, 20 mM glucose, 0.2 mM EDTA, and 0.1M KCl) which was used for determination of membrane potential [11]. For assay by inhibition zone, five ml of low melting agar (1%) mixed with 100 μl of overnight-cultured bacteria was spread on 1% regular agar plate. Two μl of diluted TP4-derived peptides (0.5 and 2 μg/μl each, inside and outside, respectively) were dotted on the top layer of microbe-containing agar plates and incubated overnight at 30/37^o^ C.

### Assays of membrane potential and permeability

*P*. *aeruginosa* PAO1 cells were washed in buffer (5 mM Hepes, pH 7.2, and 20 mM glucose), and re-suspended in the same buffer (2x10^7^ cfu/200 μl) with the addition of 0.2 mM EDTA. The bacteria were incubated with 0.4 μM DiSC3(5) in the dark for 2 hr at room temperature with gentle agitation (150 rpm). The osmotic gradient was equilibrated to a final concentration of 0.1 M KCl. The TP4 peptides were added to the above-mentioned cell suspension in a High Precision Cell cuvette (Hellma Analytics, Mulheim, Germany). The fluorescence intensity was determined by an FP-8500 fluorescence spectrophotometer (Jasco, Tokyo, Japan) with an excitation wavelength of 622 nm and an emission wavelength of 670 nm [11]. For determination of permeability, bacteria were washed and suspended in distilled water (2–5 x10^7^ cfu/100 μl) and incubated with 1 μM SYTOX^®^ Green (Molecular Probes) in a 96-well microplate for 5 min in the dark before addition of TP4. The fluorescence intensity of SYTOX^R^ Green bound to cytosolic DNA was determined by a SpectraMax M2 microplate reader (Molecular Devices, CA, USA) with an excitation wavelength of 485 nm and an emission wavelength of 520 nm [12].

### Measurement of ANS fluorescence

The emission spectra of 8-anilino-1-naphthalenesulfonate (ANS) excited at 380 nm were measured between 400 and 600 nm at 20°C using a temperature-controlled spectrofluorometer (FP-8500 Jasco, Japan) [13]. A small volume of ANS stock was stepwise added to a 200-μl solution containing 4 μg TP4 and sarkosyl or LPS at the final concentrations of 10, 20, 30, 40 and 50 μM throughout the study.

### Minimum inhibitory and bactericidal concentrations (MIC, MBC)

All the tested microbes were cultured at 37°C except *C*. *albicans* (30°C) in respective medium. 90 μl of cell suspension (2–4 x10^6^ cfu/ml) mixed with 10 μl of diluted TP4 were loaded in 96-well microplate and incubated overnight with gentle agitation at 100 rpm. The absorbance of TP4-treated bacteria in 96-well plate was measured by SpectraMax 190 microplate reader at 600 nm. The remaining cfu in the TP4-treated sample was determined by plating on agar plates. Minimum inhibitory concentration (MIC) was defined as the lowest AMP concentration in order to keep the solution with the same absorbance as that of culture medium. Minimum bactericidal concentration (MBC) was defined as the lowest AMP concentration able to kill 99.99% of microorganisms [14]. At least three independent experiments were performed to determine the average value with standard deviation.

### Inhibition and eradication of biofilm

The formation of biofilm was performed according to procedures by Stepanovic [15] with minor modifications. Overnight culture of MRSA was washed and re-suspended in TSB broth containing 1% glucose (2–4 x10^6^ cfu/ml). The bacterial solution (90 μl) mixed with 10 μl control or diluted TP4 was loaded onto 96-well microplates and incubated overnight at 37°C with 100 rpm agitation for biofilm formation. For biofilm eradication, the preformed biofilm was washed by 150 μl phosphate-buffered saline (PBS) (137 mM NaCl, 2.7 mM KCl, 10 mM sodium phosphate, pH7.5) three times and treated with TP4 overnight. The planktonic cells were removed and the adherent biofilm was washed three times with PBS. One hundred and twenty-five μl 0.1% (w/v) crystal violet was added and incubated at room temperature for 30 min. Excess crystal violet was removed and washed three times with water. The crystal violet remaining in the biofilm were resolved in 125 μl 95% ethanol and OD_600_ was measured by SpectraMax 190 microplate reader. These experiments were done in triplicate.

### Hemolytic assay

For blood collection, cardiac puncture was performed on anesthetized mice (by 3% isoflurane) by a 1 ml heparin-rinsed syringe with a 22 gauge needle. After blood collection, the animals were euthanized by cervical dislocation. Fresh mouse blood was rinsed three times with PBS, centrifuged for 5 minutes at 1500 rpm and re-suspended in PBS (1:30 dilution). The diluted erythrocyte (150 μl) was incubated with TP4-derived peptides at 37°C for 3 hr and centrifuged at 120x g for 5 minutes. The supernatants (100 μl) were transferred to a 96-well plate and the absorbance of released hemoglobin was measured by SpectraMax 190 microplate reader at 414 nm. Percent hemolysis was calculated by the following formula: % hemolysis = [(A414_in the peptide solution_−A414_in PBS_)/(A414_in 0.1% Triton-X 100_ –A414_in PBS_)]. Zero and 100% hemolysis were determined in PBS and 0.1% Triton-X 100, respectively [16].

### Binding of recombinant OprI to biotinylated AMPs

Streptavidin-conjugated beads were incubated with biotinylated TP4 peptide in PC buffer (20mM Hepes, pH 7.4, 0.05 M NaCl), or sarkosyl solution (10 mM sodium phosphate, pH 7.4, 0.1 M NaCl, 0.075% sarkosyl) for 2 hr at 4°C on a rolling wheel. The immobilized and biotinylated TP4 peptide was further mixed with recombinant OprI overnight at 4°C, washed three times with respective buffer and subjected to non-reducing SDS-PAGE/Coomassie blue staining. The preparation of recombinant OprI was described as mentioned previously [17].

### Circular dichroism (CD) spectroscopy

TP4 (60 μM) was prepared in sarkosyl solution or PC buffer for CD experiments. Alternatively, CD spectrum was also performed in PBS containing 25 mM SDS or 15 mM DPC. The CD spectra were acquired with an Aviv CD 202 spectrometer (Lakewood, NJ) and recorded at 25°C with wavelength ranges between 260 and 200 nm using a 1-mm path length quartz cuvette. All spectra were averaged over three scans and converted to mean residue ellipticity [θ] [18].

### NMR spectroscopy

Each NMR sample contained 0.25 ~ 0.35 mL of 1.5 mM peptide with 100 mM dodecylphosphocholine-d_38_ in 20 mM sodium phosphate buffer at pH 3.5 in a Shigemi tube. All NMR spectra were acquired on Bruker AVANCE 600 MHz spectrometers equipped with a triple (^1^H, ^13^C and ^15^N) resonance cryoprobe with a shield z-gradient. Two-dimensional experiments, including total correlation spectroscopy (TOCSY) with two different mixing times of 70 and 100 ms, nuclear Overhauser effect spectroscopy (NOESY) at two mixing times of 150 and 300 ms, and double-quantum filtered correlation spectroscopy (DQF-COSY) were performed at 318 K. Solvent suppression was performed by using pre-saturation, watergate W5 or 3-9-19 pulse sequences with gradients. TOCSY and NOESY experiments were collected into 2048 points in *t*_2_ dimension and 320 points in *t*_1_ dimension with 24 or 88 scans per *t*_1_ increment at a relaxation delay of 1.5 s between each scan. For DQF-COSY experiments, 4096 and 256 data points were collected and zero-filled to 8K and 512 points in *t*_2_ and *t*_1_ dimensions. The ^3^JHN_α_ coupling constants were measured from DQF-COSY spectra with a spectral resolution of 0.73 Hz per point in *t*_2_ dimension by using the method of Kim and Prestegard [19]. Intramolecular hydrogen bonds were estimated by using temperature coefficients calculated from a series of TOCSY spectra at 298, 303, 308, 313 and 318 K as well as HD exchanges from visible NH-C_α_H cross peaks of TOCSY spectra in the time intervals after sample had been dissolved in D_2_O. All of the NMR spectra were processed using Bruker TopSpin 3.1 and analyzed using Sparky (Goddard, T.D. and Kneller, D.G. University of California, San Francisco).

### Structural calculations and paramagnetic relaxation enhancement (PRE)

NMR structures of TP4 were calculated and refined from all experimental restraints by dynamically simulated annealing procedures in the Xplor-NIH program [20]. Distance restraints were acquired from NOEs assigned in NOESY spectra with the mixing time of 150 and 300 ms. The NOE cross-peak intensities were classified as strong, medium, and weak, while corresponding to distance limits of 1.8 to 2.5 Å, 1.8 to 3.5 Å, and 1.8 to 6.0 Å, respectively. All force constants and molecular parameters were set to default values. Simulated annealing was performed using 30,000 steps at 1,000 K and 30,000 steps as the molecules were gradually cooled to 100 K. The 200 calculated structures were refined using the SA refinement “refine.inp”. The temperature in refine.inp was set to 1,000 K and gradually cooled to 100 K in 60,000 steps. In this protocol, the final van der Waals radii in the cooling step was increased ($fin_rad = 0.80, and the original value is 0.75) to reduce the too-close contacts between heavy atoms. The 15 refined structures with the lowest energies, no distance constraint violations greater than 0.3 Å and no dihedral angle constraint violations greater than 5 degrees, were selected for further analysis. These structures were depicted and analyzed by PyMOL (https://www.pymol.org), MOLMOL and PROCHECK-NMR [21,22]. Different concentrations of MnCl_2_ (0.5, 1.0, and 2.0 mM) were added to NMR samples (1.5 mM TP4 with 100 mM DPC micelles in 20 mM sodium phosphate buffer, pH3.5). The effects of MnCl_2_ were determined by comparing the intensities of H_N_-H_α_ cross-peaks in TOCSY spectra with and without MnCl_2_ at 318K.

### Data bank accession number

The chemical shifts of TP4 at pH 3.5 and 318 K have been deposited in the BioMagResBank (accession number BMRB-36025), and the best 15 structures of TP4 have been deposited in the RCSB Protein Data Bank (accession number 5H2S).

## Results

### Effect of TP4 on the viability, membrane potential and permeability of bacteria

**Fig 1:**
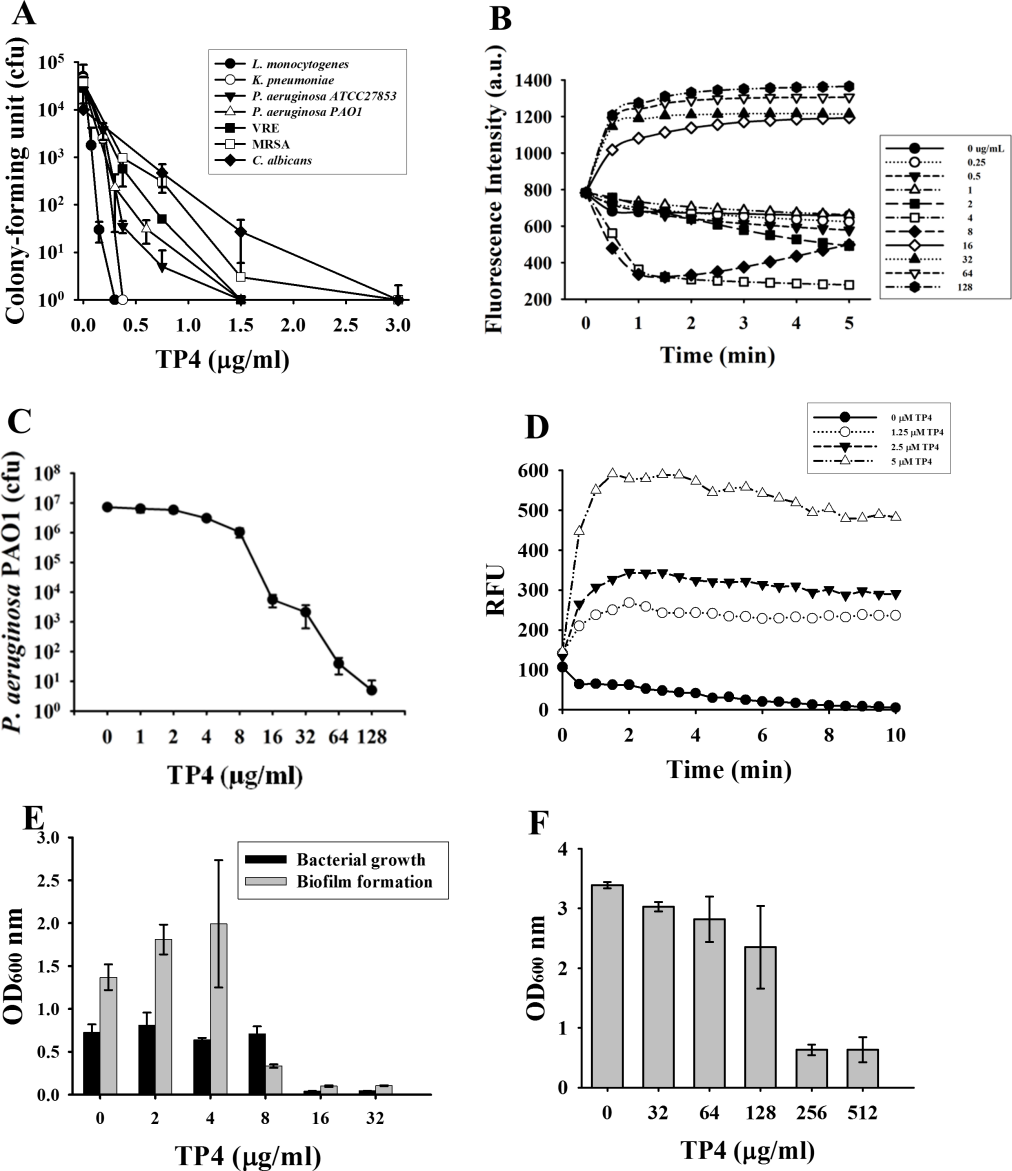
Bactericidal activities of TP4. (A) Antimicrobial spectrum of TP4. Small aliquots of microbes (5-10x10^4^ cfu/45 μl) were incubated with diluted TP4 (5μl) in phosphate buffer at 37°C for 3 hr and plated for the determination of the remaining cfu. MRSA and VRE represent methicillin-resistant *Staphylococcus aureus and* vancomycin-resistant *Enterococcus*. (B) Membrane potential of TP4-treated *P*. *aeruginosa*. Fluorescence intensity of DiSC3 was monitored using 622 nm and 670nm as excitation and emission wavelength. Data plotted are representative average values of three independent trials. (C) Bactericidal activity of TP4 against *P*. *aeruginosa* PAO1. Small aliquots of microbes (5 x10^6^ cfu/45μl) was suspended in the membrane potential assay buffer of panel B for the determination of remaining cfu. (D) Membrane permeability of *P*. *aeruginosa* PAO1. The fluorescence of SYTOX^TM^ Green was monitored using 485 nm and 520 nm as excitation and emission wavelength. RFU, relative fluorescence unit. Data plotted are normalized with values of untreated samples and representative average values of three independent trials. (E) Inhibition of biofilm formation. MRSA (2–4 x10^6^ cfu/ml) mixed with diluted TP4 in TSB broth containing 1% glucose were incubated at 37°C with 100 rpm shaking overnight. Biofilm formation after crystal violet staining and planktonic cell growth were quantitated at the wavelength of 600 nm. (F) Eradication of biofilm. Preformed MRSA biofilm was treated with diluted TP4 overnight. The adherent biofilm was quantified by crystal violet staining and monitored at a wavelength of 600 nm. Each experiment was performed in triplicate.

TP4 exerted a broad antimicrobial spectrum. The Gram-positive *Listeria monocytogenes* was the most susceptible among the microbes tested in 10 mM phosphate buffer, pH 7.4 (0.1 μM TP4 for 10^2^-fold reduction in cfu) and thereafter in the order of Gram-negative *Klebsiella pneumoniae*, *Pseudomonas aeruginosa* ATCC27853 and *P*. *aeruginosa* PAO1 (0.12 μM). The Gram-positive vancomycin-resistant enterococcus clinical isolate VRE (0.15 μM), *Staphylococcus aureus* MRSA (0.25 μM) and fungus *Candida albicans* (0.25 μM) were less sensitive to TP4 than the above-mentioned bacteria (Fig 1A). Similar to most antimicrobial peptides (AMPs), TP4 depolarized the membrane potential of *P*. *aeruginosa* PAO1 since the DiSC_3_(5) dye was released into the surrounding medium that caused an increase of fluorescence intensity at higher concentrations of TP4 (16 μM or above). However, hyperpolarization was observed at lower concentrations (4 μM, 8 μM) a few minutes after TP4 treatment (Fig 1B). It is worthy to note that at 8 μM it started to cause cfu reduction in the membrane potential assay buffer (Fig 1C). The membrane permeability of *P*. *aeruginosa* PAO1 increased immediately after TP4 treatment (1.25–5 μM) (Fig 1D). The biofilm formation of MRSA was markedly inhibited by TP4 at the sub-inhibitory concentration, 8 μg/ml, which did not inhibit the growth of planktonic bacteria (Fig 1E). The preformed biofilm was almost eradicated by 256 μg/ml TP4 (Fig 1F).

### Enhanced binding of TP4 to receptor protein OprI by sarkosyl and LPS

**Fig 2:**
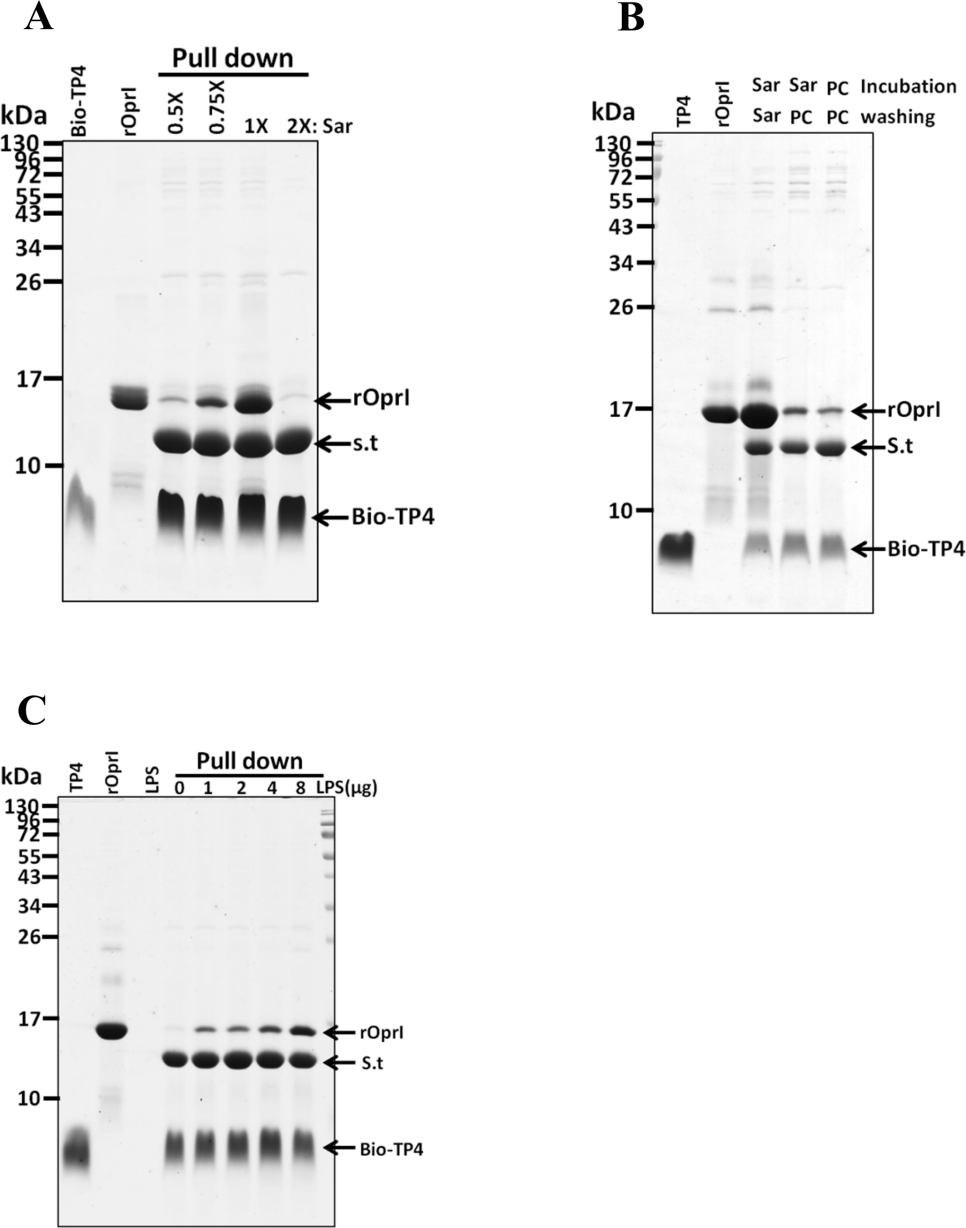
Sarkosyl- and LPS-enhanced binding of TP4 to OprI. (A) Sarkosyl-enhanced binding of TP4 to recombinant OprI (rOprI). The biotinylated TP4 (6 μg) was immobilized on streptavidin-conjugated gels, then incubated with recombinant OprI (10 μg) in different concentration of sarkosyl solution; and the pellets were subjected to non-reducing SDS-PAGE/Coomassie blue staining. (B) Reversible binding of TP4 to recombinant OprI. Biotinylated TP4 was immobilized on Streptavidin-conjugated beads, then incubated and washed with PC buffer or sarkosyl solution. The pellets were analyzed as panel A. PC, PC buffer. (C) LPS-enhanced binding of TP4 to OprI. The biotinylated TP4 (6 μg) was immobilized on Streptavidin-conjugated gels in PC buffer, then incubated with recombinant OprI in the presence of LPS as indicated. 1x Sar solution contains 0.075% sarkosyl (w/v). Bio-TP4, biotinylated TP4; S.t, Streptavidin.

Our previous studies showed that the outer membrane protein (OprI) of *P*. *aeruginosa* and the outer membrane lipoprotein (Lpp) of Enterobacteriaceae family of Gram-negative bacteria are targeted and internalized into cytosol by cationic amphipathic antimicrobial peptides [10,12]. To observe the behaviors of TP4 while it contacts with the bacterial surface, the effects of both membrane-like surfactant sarkosyl and abundant surface component lipopolysaccharide (LPS) were examined. The results showed that the OprI-binding ability of biotinylated TP4 markedly increased in the presence of 0.075% sarkosyl (2.6 mM designated as 1x Sar), but not at lower (0.5x Sar) and higher (2x Sar) concentrations (Fig 2A). However, the 1x Sar-induced OprI-TP4 complex dissociated again when sarkosyl was removed from the solution (Fig 2B). It indicates that OprI-TP4 binding is reversible depending on the concentration of sarkosyl employed. Similarly, LPS was also able to increase the OprI-binding ability of biotinylated TP4 in a dose-dependent manner (Fig 2C).

### Conformational changes of TP4 induced by sarkosyl and LPS

**Fig 3:**
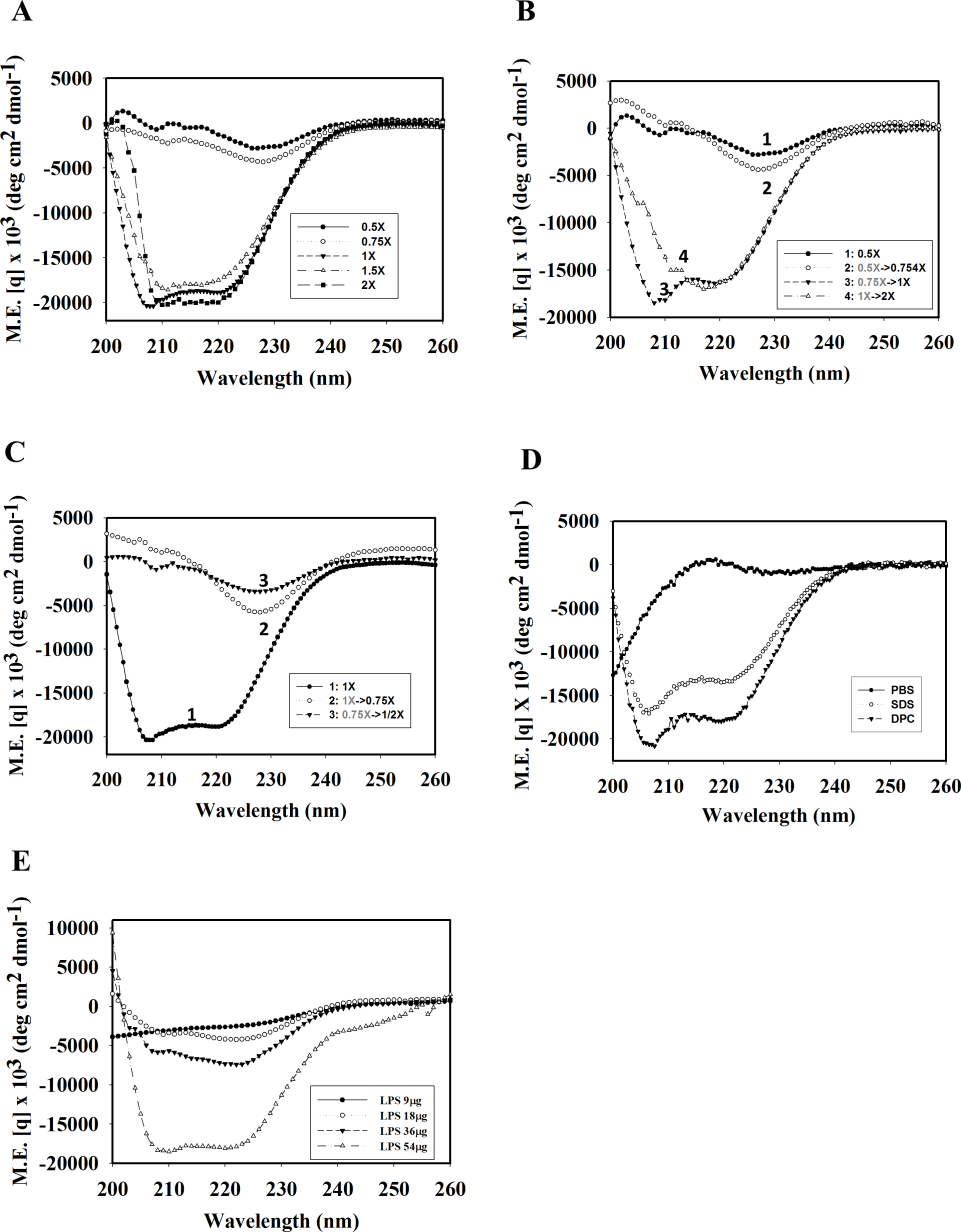
Helix formation of TP4 induced by sarkosyl and LPS. (A) CD spectra of 60μM TP4 (54μg/300μl) in various concentrations of sarkosyl solution. (B, C) Dynamic changes of the secondary structure of TP4 by sarkosyl at different concentrations. (D) CD spectra of 60μM TP4 in the presence of 25 mM SDS or 15 mM DPC in PBS. (E) CD spectra of 60μM TP4 in the presence of LPS as indicated.

**Fig 4:**
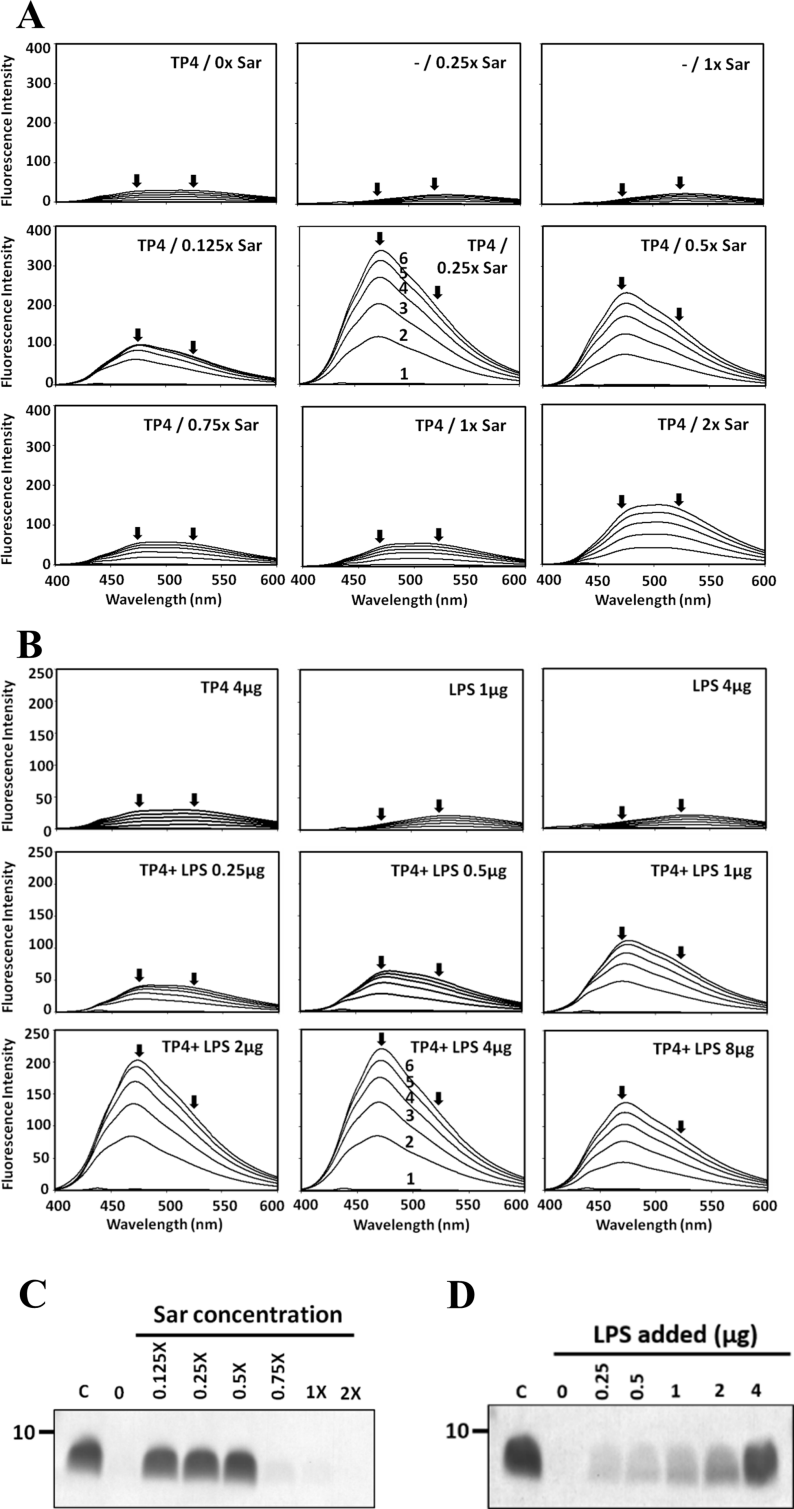
Hydrophobicity and solubility of TP4 in the presence of sarkosyl and LPS. (A) ANS emission spectrum of TP4 in solution containing sarkosyl. TP4 (4 μg) was dissolved in 200 μl of 10 mM sodium phosphate, pH7.4, 0.1 M NaCl, containing various concentrations of sarkosyl (0.075%, w/v, designated as 1x). (B) ANS emission spectrum of TP4 in solution containing LPS. TP4 (4 μg) was dissolved in 200 μl of 10 mM sodium phosphate, pH7.4, containing various amounts of LPS as indicated. ANS was added stepwise to the final concentrations as indicated (lines 1 to 6 at 0, 10, 20, 30, 40 and 50 μM, respectively). Arrows indicate the emission maximum at 470 nm or 520 nm for bound- and free-form ANS, respectively. (C, D) Solubility of TP4 in solution containing either sarkosyl or LPS. TP4 (4 μg) dissolved in 200 μl of respective solution containing sarkosyl or LPS at concentration as indicated was spun at 14,000 x *g* for 15 min and analyzed by SDS-PAGE/Coomassie blue staining.

To examine the positive effects of sarkosyl and LPS on the enhanced binding of TP4 to OprI, the secondary structures of TP4 were examined by CD spectrum analyses. The results showed that it was typically random in PC buffer or PBS. In contrast, it exhibited helical structure in membrane-like environments (2.6 mM sarkosyl [1x Sar], 25 mM SDS and 15 mM DPC) by having two local minima at 208 and 222 nm, but not at lower concentrations of sarkosyl (0.75x, 0.5x Sar) (Fig 3A and 3D). Also noted was that the non-structural TP4-containing solution (0.5x Sar) was turbid, but became clear when the sarkosyl concentration was gradually increased to 1x Sar during which α-helices formed again (Fig 3B). In contrast, the α-helix formed at 1x Sar solution diminished if the sarkosyl was stepwise diluted to 0.75x or 0.5x Sar (Fig 3C). Furthermore, the non-structural TP4 was also converted to α-helix by LPS in a dose-dependent manner (Fig 3E). To further examine the conformational changes of TP4 attributed to sarkosyl and LPS, hydrophobicity was analyzed by ANS fluorescence assay [13]. ANS probes the hydrophobic regions of peptide/protein. The free form ANS exhibits an emission maximum at 520 nm while the bound form ANS mainly at 470 nm (blue shift). TP4 underwent a blue shift from 520 to 470 nm in 0.125 to 0.5x Sar solution, in which no helical structures were observed (Fig 4A). Interestingly, the majority of TP4 precipitated in 0.125~0.5x Sar, but not in 1x or 2x Sar after high speed centrifugation (14,000 x *g*, 15 min) (Fig 4C). For LPS, the blue shift was clearly seen at the LPS/TP4 weight ratio from 0.25 to 2.0 (Fig 4B) and the LPS-driven α-helical TP4 was pulled down in a dose-dependent manner after centrifugation (Fig 4D). These results suggest that 1.0x Sar can drive TP4 into a conformational change from a non-structure to an α-helical structure, and that LPS can also drive TP4 to an α-helical structure but with high hydrophobicity and low solubility.

### NMR resonance assignment and solution structure of TP4

**Fig 5:**
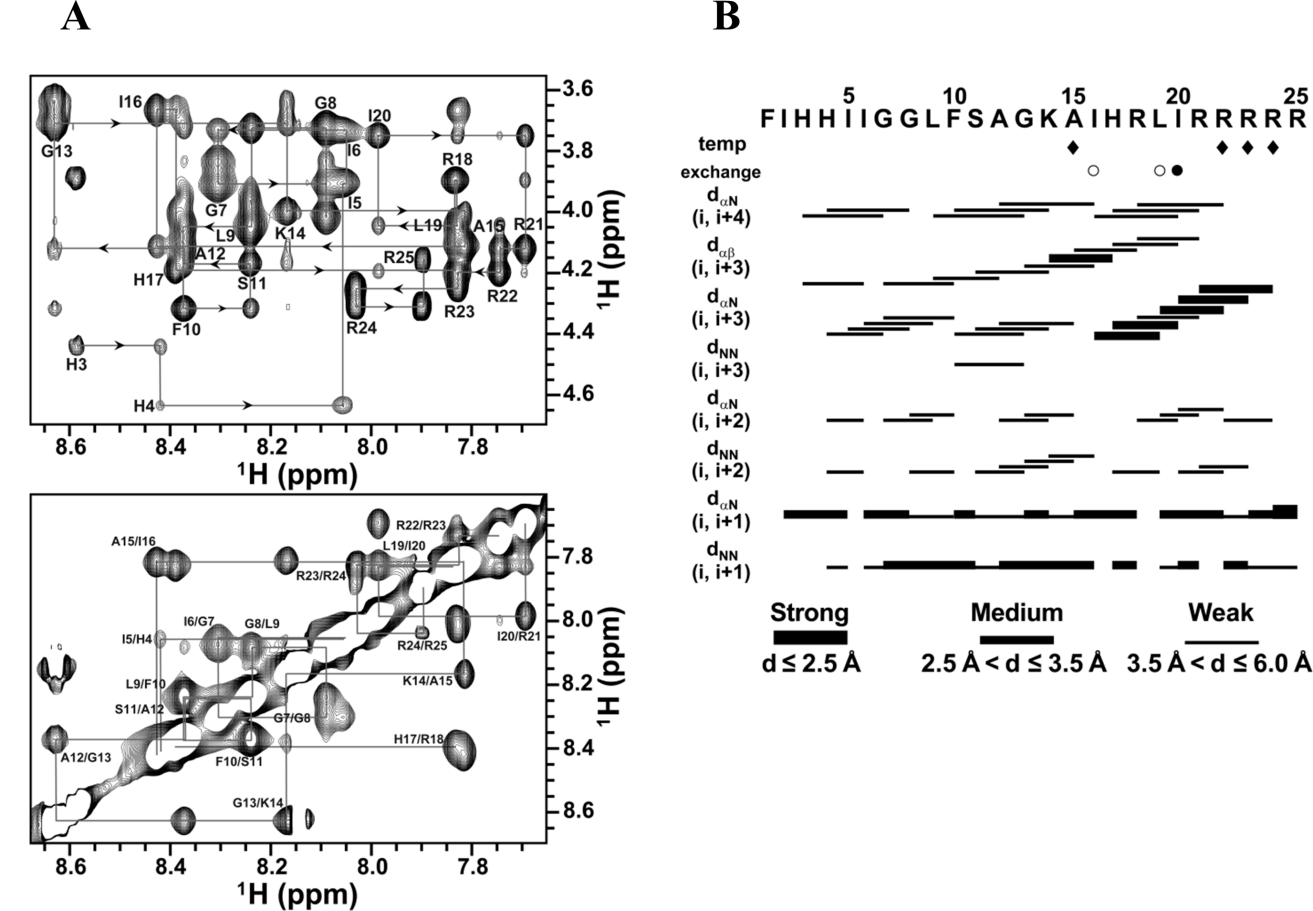
NMR spectra and NOE connectivity of TP4 bound to DPC micelles. (A) The fingerprint (the upper panel) and amide-amide (the lower panel) regions of NOESY spectrum recorded at 150 ms mixing time for TP4 in DPC micelles at pH 3.5 and 318 K. Sequential resonance assignments of TP4 are labeled at the positions of NH-C_α_H and NH-NH cross-peaks. (B) Summary of NOE connectivity, amide proton exchange, and temperature coefficients for TP4. Opened and filled circles in the exchange row show that the exchangeable amide protons are still visible after 6 hr and 12 hr in D2O. The temperature coefficient values greater than -4.5 ppb/K are shown as filled diamonds. The thickness of the line is relative to the intensity of the NOE as indicated in the bottom.

**Fig 6:**
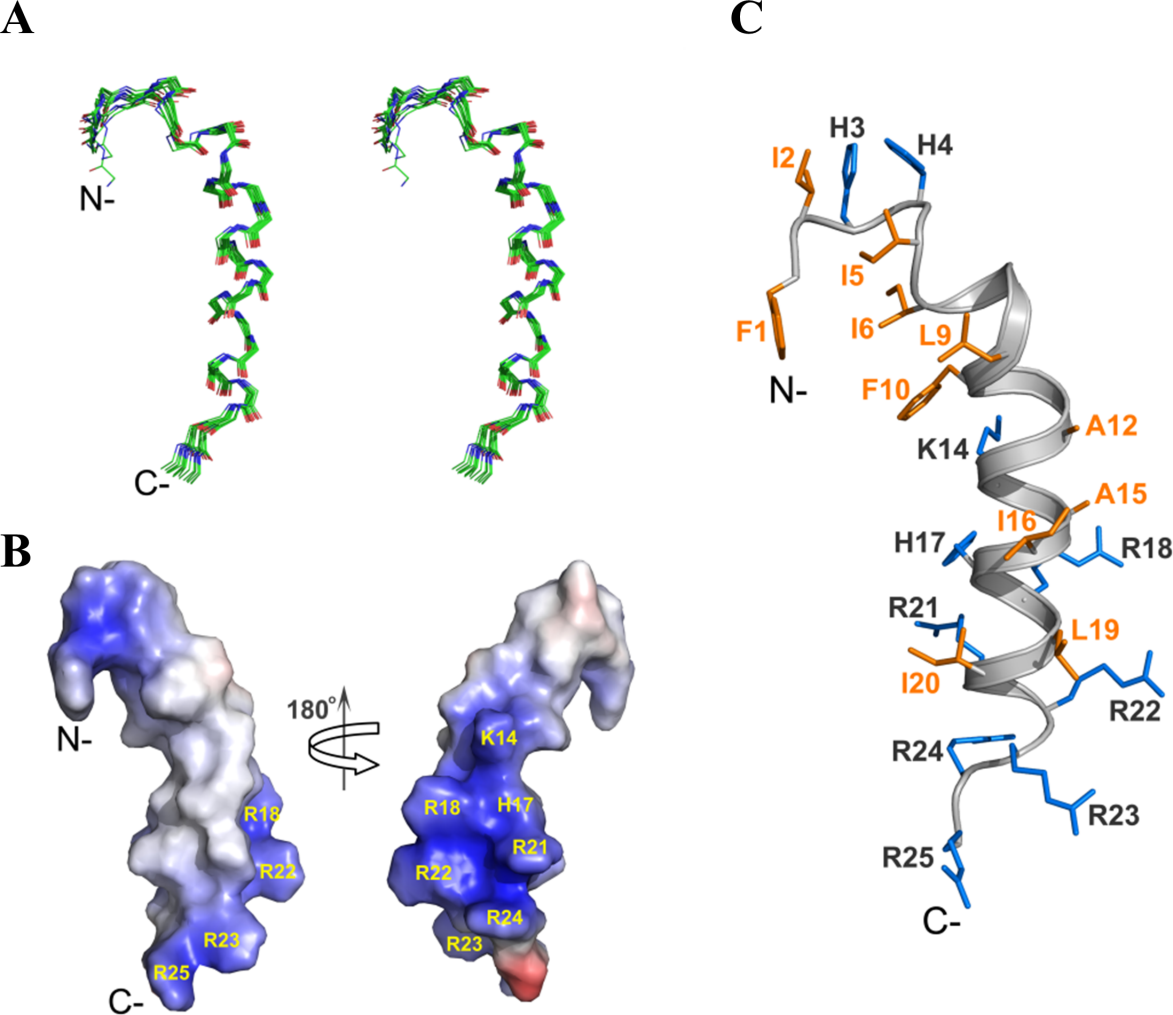
Solution structure of TP4 in DPC micelles. (A) The backbone atoms of the 15 lowest energy structures of TP4 are superimposed and shown in stereo view. Atoms of carbon, nitrogen and oxygen are colored in green, blue and red, respectively. (B) The electrostatic surface plot of TP4 is colored by vacuum electrostatic functions of PyMOL. Positive, negative, and neutral charges are indicated by blue, red and white colors respectively. (C) The lowest energy structure of TP4 bound to DPC micelles is displayed. The side chains of the hydrophobic and positive residues are colored in orange and marine. The orientations of TP4 in (B) and (C) are the same as (A).

Although the CD spectra showed that TP4 forms an α-helix in these membrane-mimicking environments (sarkosyl, SDS and DPC), TP4 gave well-dispersed NMR signals only in DPC micelles. NMR resonances were mostly assigned except for Phe1 and H_N_ of Ile2 based on 2D-^1^H NMR spectra (Fig 5A**)**. A total of 359 NOE distance restraints including 159 intra-residues, 90 sequential, and 110 medium-range as well as 16 backbone dihedral angles, were used in structure calculation (Table 1). Both the temperature coefficients and H/D exchange rates of the backbone amide protons were analyzed to derive H-bond restraints; that is, an amide proton with a slow exchange H/D rate or a temperature coefficient greater than -4.5 ppb/K [23]. The NOE connectivity of some residues, such as Ala15, was not complete due to peak overlap and ambiguity. In segment containing Ile6-Phe10, few NOEs followed the correlations of *d*_*αβ*_ (*i*, *i*+3) and *d*_*α*N_ (*i*, *i*+4) and only two hydrogen bonds were observed between oxygen atoms of Ile6 and H_N_ of Leu9 or Phe10. While numbers of *d*_*α*N_ (*i*, *i*+3), *d*_*αβ*_ (*i*, *i*+3), and *d*_*α*N_ (*i*, *i*+4), which are the characteristics of α-helical NOEs were observed from Phe10 to Arg22 (Fig 5B). The ensemble of 15 lowest energy structures (Fig 6A) with average root mean squared deviations (RMSD) of 0.423 Å for the backbone atoms (N, C_α_, C’ and O) and 0.967 Å for all heavy atoms was generated and the structural statistics are shown in Table 1. TP4 in DPC micelles assumes a typical α-helix at residues Phe10-Arg22 and a distorted helical segment at Ile6-Phe10 (Fig 6C) with the average bent angle of ~22.6° between these two helical segments. The five C-terminal Arg residues were shown to cluster together to form a hydrophilic patch and the five hydrophobic residues at the N-terminus, Phe1, Ile5, Ile6, Leu9 and Phe10 apparently form a hydrophobic core (Fig 6B and 6C). The orientation of TP4 in DPC micelles was determined by PRE experiments. In low concentrations of Mn^2+^, most intensities of H_N_-C_α_H cross-peak remain the same except for Arg25 which shows lower than 0.5 in intensity retention. By contrast, in higher Mn^2+^ concentration (2 mM), cross-peak intensities of Arg23-Arg25 and His4 were dramatically reduced (S1 Fig). These findings suggest that Arg25 is located in the aqueous phase while Arg23, Arg24 and His4 are likely located near the head group of micelles.

**Table 1:** Structural statistics of TP4 in DPC micelles at pH 3.5 and 318 K.

**Total no. of restraints**	
**Intraresidual**	**159**
**Sequential (|i-j| = 1)**	**90**
**Medium range (|i-j| < 5)**	**110**
**Long range (|i-j| ≥ 5)**	**0**
**Dihedral angle**	**16**
**Atomic RMSD**	
**Backbone atoms (Å)**	**0.423**
**All heavy atoms (Å)**	**0.967**
**Deviations from idealized geometry**	
**Bonds (Å)**	**0.004 ± 0.000**
**Angles (deg)**	**0.483 ± 0.008**
**Impropers (deg)**	**0.358 ± 0.008**
**Ramachandran plot**	
**Most favored (%)**	**73.3**
**Additionally allowed (%)**	**24.7**
**Generously allowed (%)**	**2.0**
**Disallowed (%)**	**0.0**

### Residues for bactericidal activity

**Fig 7:**
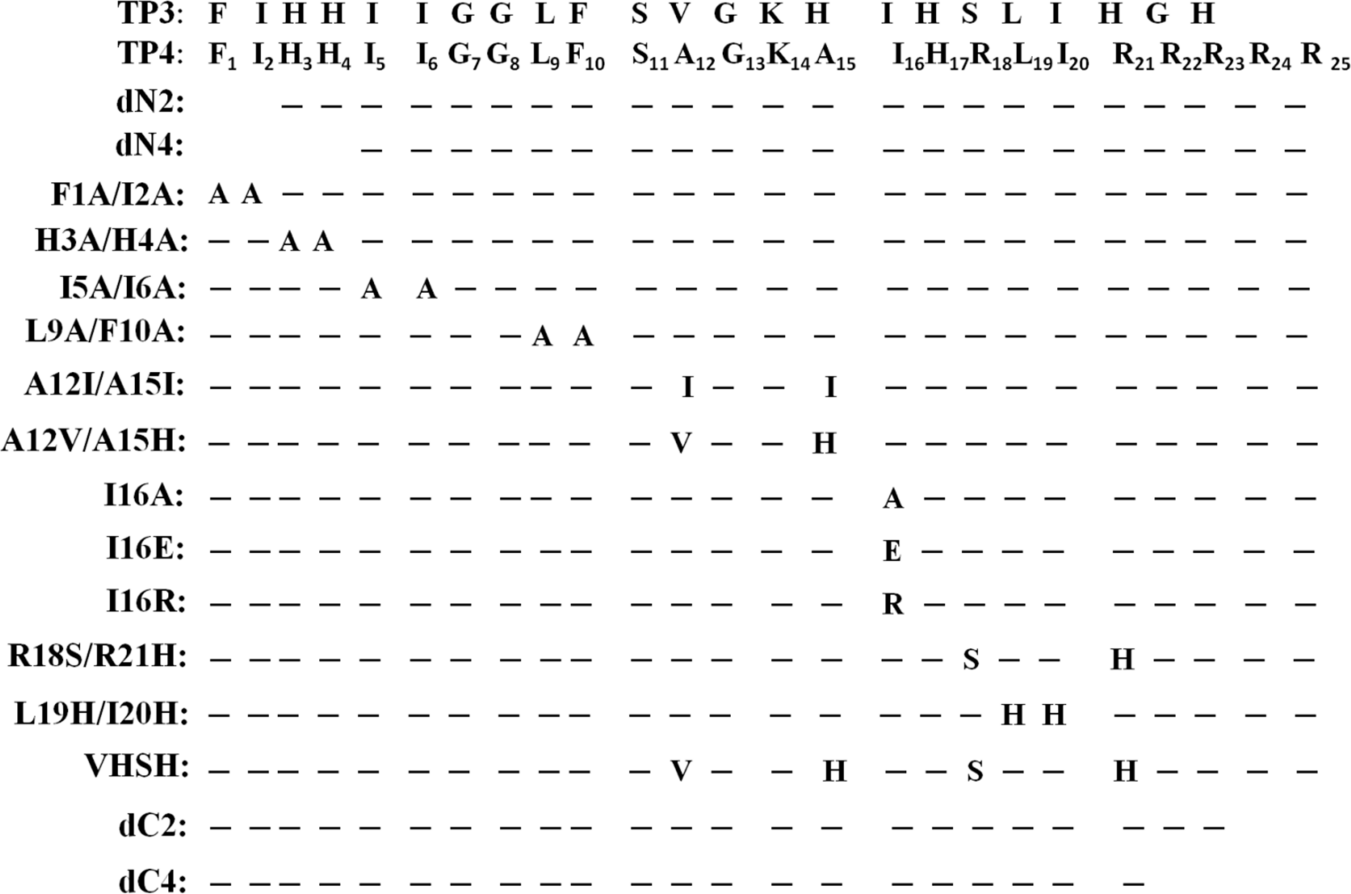
Amino acid sequences of TP3 and TP4-derived peptides. Dashed line indicates the residue identical to that of wild type TP4.

To determine the residues responsible for these characteristic properties of TP4, like structure, hemolytic and bactericidal activity, some hydrophobic and cationic residues were deleted or substituted with alanine or corresponding residues of a homologous peptide such as TP3, which possesses less activity than TP4 as demonstrated in Fig 7. First, the antimicrobial activities against Gram-positive, -negative bacteria and fungi were examined by inhibition zone assay on agar plates as shown in S2 Fig. For *P*. *aeruginosa* PAO1, the C-terminal arginine deletion (dC4), L9A/F10A as well as I16E substitutions abolished most bactericidal activities. With respect to *S*. *aureus*, dC4 deletion retained the activity while I16E substitution lost the activity. I5A/I6A, L9A/F10A and I16R substitutions retained partial activity. For *S*. *aureus* MRSA, the dN2 deletion and I5A/I6A, L9A/F10A, I16R, I16E, almost induced a lost in activity while dN4 deletion and H3A/H4A, I16A substitutions partially decreased the activity. It is interesting that the H3A/H4A substitution was detrimental to all tested bacteria, but non-fungicidal to *C*. *albicans* which were susceptible to all other TP4-derived peptides tested so far (S3 Fig bottom panel). It is of note that further deletion of H3/H4 residues (dN4) partially restored the lost bactericidal activity of dN2 against tested bacteria. The antimicrobial activities of these mutated peptides against various microbes in planktonic solutions were also determined by the optical density of bacteria and presented as values of MIC and MBC in Table 2. They are quite similar to those observed by the inhibition zone assay on agar plate. These results suggest that residues in TP4 responsible for antimicrobial activity against Gram-positive, -negative bacteria and fungi are different from each other.

**Table 2:** MIC and MBC of TP4 mutants against microbes (μg/ml).

AMP	*P*. *aeruginosa*		*S*. *aureus*		MRSA		*C*. *albicans*	
	MIC	MBC	MIC	MBC	MIC	MBC	MIC	MBC
**TP4**	**64**	**128**	**8**	**16**	**16**	**32**	**128**	**128**
**F1A/I2A**	**256**	**256**	**128**	**256**	**>512**	**>512**	**64**	**128**
**H3A/H4A**	**>512**	**>512**	**32**	**32**	**128**	**256**	**512**	**512**
**I5A/I6A**	**512**	**512**	**>512**	**>512**	**>512**	**>512**	**128**	**128**
**L9A/F10A**	**>512**	**>512**	**>512**	**>512**	**>512**	**>512**	**128**	**128**
**I16R**	**>512**	**>512**	**>512**	**>512**	**>512**	**>512**	**128**	**256**
**I16E**	**>512**	**>512**	**>512**	**>512**	**>512**	**>512**	**128**	**128**
**I16A**	**256**	**>512**	**64**	**128**	**64**	**512**	**128**	**128**
**L19H/I20H**	**256**	**>512**	**128**	**512**	**>512**	**>512**	**128**	**128**
**dN2**	**512**	**>512**	**512**	**512**	**>512**	**>512**	**128**	**128**
**dN4**	**256**	**>512**	**128**	**128**	**64**	**256**	**64**	**128**
**dC2**	**512**	**>512**	**16**	**32**	**32**	**32**	**128**	**128**
**dC4**	**>512**	**>512**	**64**	**128**	**256**	**256**	**128**	**256**
**A12V/A15H**	**128**	**128**	**16**	**16**	**32**	**64**	**128**	**128**
**A12I/A15I**	**512**	**>512**	**16**	**64**	**64**	**256**	**128**	**128**
**R18S/R21H**	**256**	**512**	**32**	**64**	**32**	**32**	**256**	**512**
**VHSH**	**>512**	**>512**	**16**	**16**	**16**	**32**	**128**	**128**
**TP3**	**>512**	**>512**	**256**	**256**	**256**	**>512**	**128**	**128**

### Residues involved in hemolytic activity

**Fig 8:**
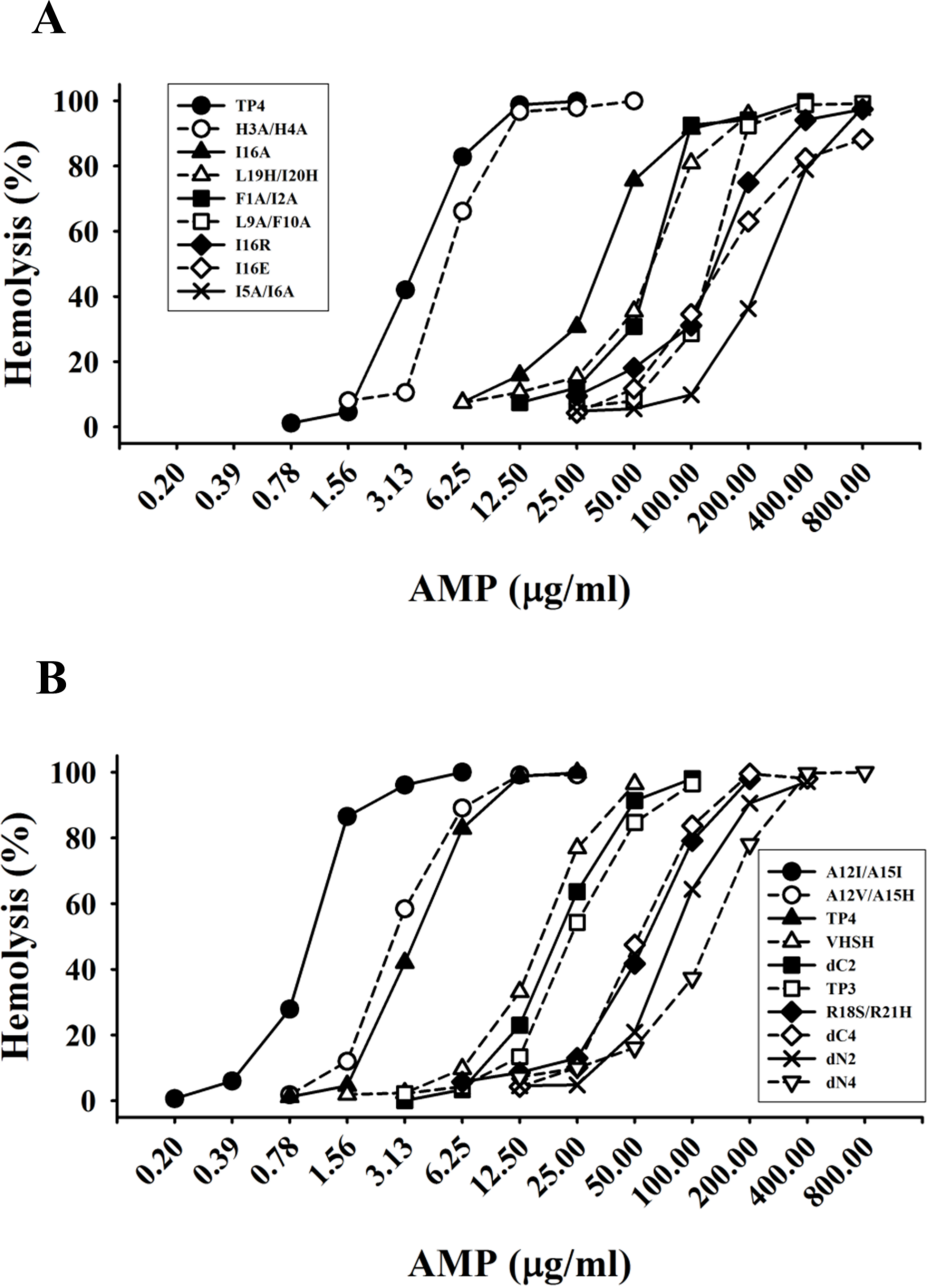
Hemolytic activity of TP4-derived peptides. Diluted mouse red blood cells (150 μl) was incubated with TP4-derived peptides at 37°C for 3 hr and spun at 120 x *g* for 5 minutes. The supernatants (100 μl) were transferred to a 96-well plate for measurement of released hemoglobulin at 414 nm by the SpectraMax 190 microplate reader.

To determine the potential of TP4 as a therapeutic agent to treat infection caused by drug-resistant bacteria, the hemolytic activity towards mouse red blood cells was examined. H3A/H4A and A12V/A15H peptides showed similar hemolytic activities as that of wild type TP4 while A12I/A15I exhibited four times higher activity than wild type TP4. Interestingly, peptides lacking N-terminal hydrophobic residues such as Phe1, Ile2, Ile5, Ile6, Leu9, Phe10, and C-terminal arginine residues such as Arg18, Arg21-Arg25, dramatically lost their hemolytic abilities (Fig 8A and 8B). In regards to the hydrophobic residues located on the hydrophobic face of the main helix, Ile16 and Leu19/Ile20, were also shown to be critical for hemolytic activity. These results suggest that both hydrophobic and cationic residues play important roles in hemolytic activity.

### Residues for bacteria- and OprI-binding

**Fig 9:**
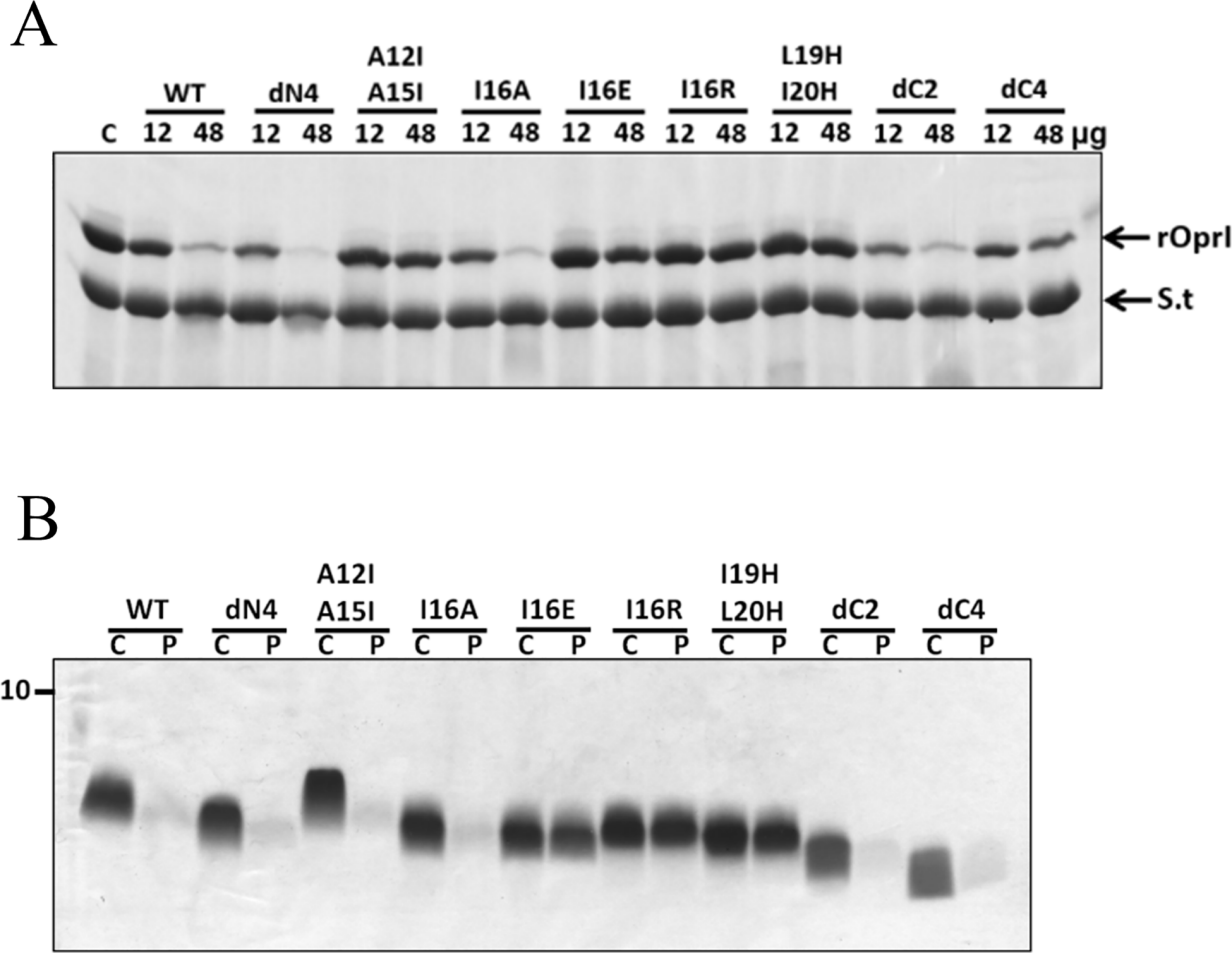
OprI-binding ability of TP4-derived peptides. **(A)** Competition for OprI binding by TP4-derived peptides. Recombinant OprI (12μg, 48μg each) was pre-incubated with free mutated peptide before binding to biotinylated-TP4 which was immobilized on Streptavidin-conjugated gel. The gel pellet was analyzed by non-reducing SDS-PAGE/Coomassie blue staining. rOprI, recombinant OprI; S.t, Streptavidin. (B) Solubility of TP4-derived peptide. TP4-derived peptides (6 μg each) dissolved in 1x sarkosyl buffer (300 μl) were incubated at 37°C for 10 min, then spun at 14,000 x *g* for 15 min and analyzed by SDS-PAGE/Coomassie blue staining. C, control without treatment; P, precipitation.

To determine key residues responsible for bacterial binding, TP4-derived peptides were incubated with both Gram-negative *P*. *aeruginosa* PAO1 and Gram-positive *S*. *aureus*. This was followed by pull down of the bacteria and SDS-PAGE analysis. Deletion of C-terminal arginines (dC2 and dC4) markedly reduced the binding ability to both Gram-positive and -negative bacteria while no significant reduction was observed for the other peptides even if they were not bactericidal such as I16E and I16R substitutions (S3 Fig). These results indicate that positively charged residues of TP4 are helpful for its binding to bacteria probably through negatively charged surface components like LPS, although it is not sufficient for bactericidal activity. To further examine the residues responsible for OprI-binding, recombinant OprI was pre-incubated with excess amounts of mutated peptides before binding to biotinylated TP4 immobilized on Streptavidin-conjugated gel. The results showed the binding of recombinant OprI to biotinylated TP4 to be mostly competed by TP4-derived peptides in a dose-dependent manner with the exception of A12I/A15I, I16R, I16E, L19H/I20H substitutions and dC4 deletions (Fig 9A). These results indicate that only the hydrophobic residues residing on the hydrophobic face of the main helix, Ala12, Ala15, Ile16, Leu19 and Ile20, as well as C-terminal arginine clusters of TP4 are critical for OprI-binding.

### Residues for helix formation and hydrophobicity

**Fig 10:**
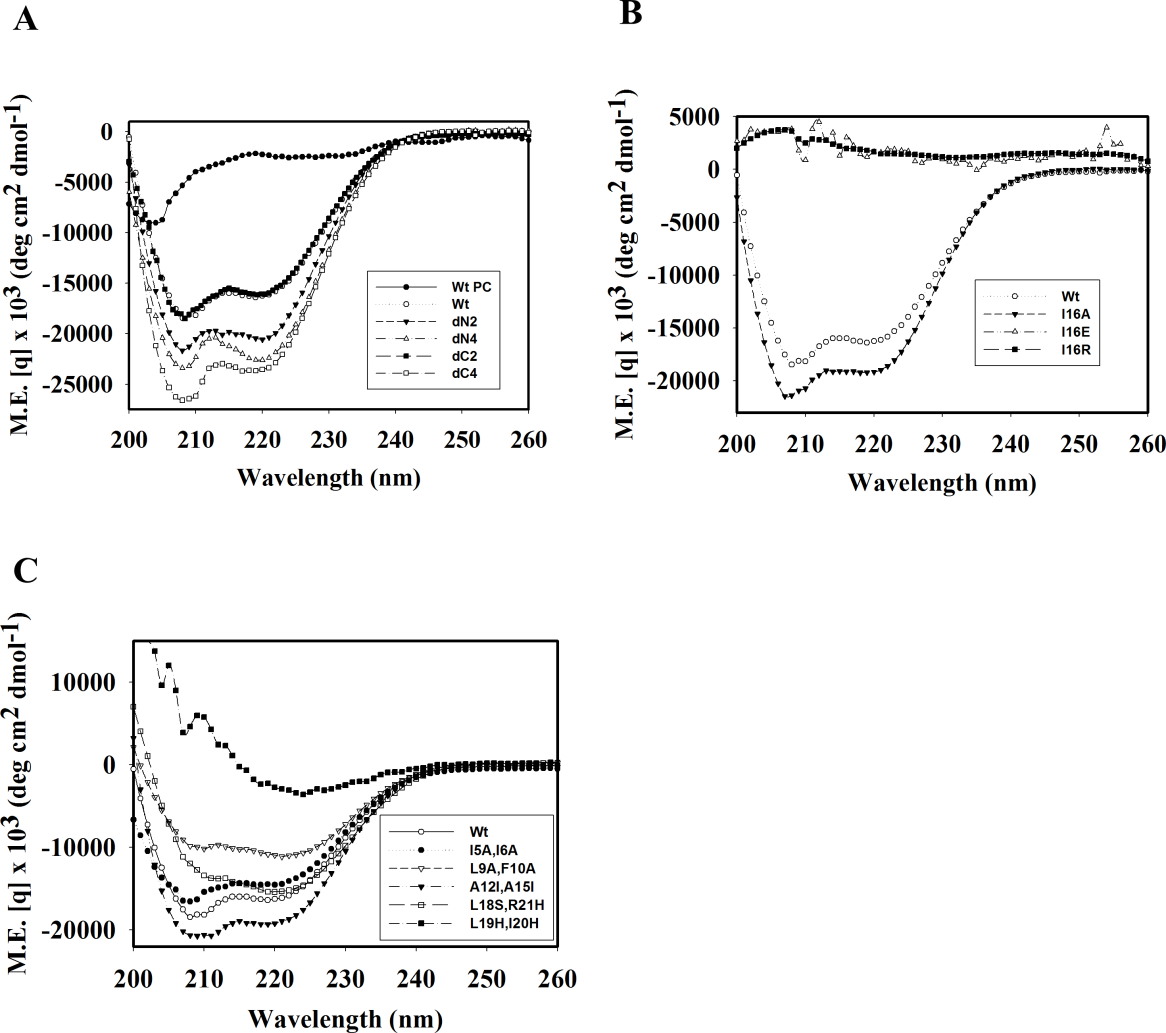
Analyses of secondary structure of TP4-derived peptides. 54 μg of TP4-derived peptides dissolved in 1x Sar buffer (300 μl) were subjected to CD spectrum analyses. Wt PC, wild-type TP4 dissolved in PC buffer.

To elucidate the effects of hydrophobic residues and arginine clusters on hemolytic and bactericidal activities due to peptide structure, CD spectrum analyses of TP4-derived peptides were performed in 1x Sar solution. Most peptides retained helical structure including N-, C-terminal deletions (dN2, dN4, dC2 and dC4), I5A/I6A, A12I/A15I and L18S/R21H substitutions (Fig 10). However, I16R, I16E and L19H/I20H substitutions caused a loss in helical structure (Fig 10B and 10C). It is of note that the signals of double minimum of L9A/F10A was weaker than that of wild type TP4, while those of dN2, dN4, dC4, A12I/A15I were stronger than wild type. Furthermore, ANS spectrum analyses showed that I16E, I16R and L19H/I20H substituted peptides exerted a markedly blue shift from 520 nm to 470 nm in 1x Sar. The spectra also showed slight shifts in dN4 and A12I/A15I, while the A12V/A15H, I16A, dC4 as well as the wild type TP4 peptides did not exert blue shift (S4 Fig). Interestingly, I16R, I16E, and L19A/I20A peptides having a markedly blue shift in the ANS emission spectrum precipitated after high speed centrifugation (14,000 x *g*, 15 min), while I16A, A12I/A15I and all other peptides did not (Fig 9B). These results demonstrate that residues residing on the hydrophobic face of the main helix, Ala12, Ala15, Ile16, Leu19 and Ile20, are critical for the integrity of the amphipathic TP4 structure which is essential for bactericidal and hemolytic activities.

## Discussion

Although AMPs possess diverse secondary structures, their surfaces are uniformly amphipathic with cationic and hydrophobic residues on opposite sides [24–26]. The former property promotes selectivity for negatively charged components on microbial surfaces like lipopolysaccharides, whereas the latter facilitates interaction with hydrophobic components on the bacterial membrane. For elucidation of action mechanisms of AMPs, various targets have been extensively proposed and studied such as the outer surface lipid, outer membrane protein, inner membrane, inner membrane protein, and even intracellular components like nucleic acids [27–29]. In addition to the well-known bactericidal activity, TP4 also exerts hemolytic and cytotoxic activity, stimulates cell proliferation and suppresses immune response in mouse models [7–9]. Therefore, determination of the TP4 structure and identification of residues responsible for these activities are helpful for the understanding of multiple functional AMPs and potential application in clinical therapy for microbial infection.

First, we solved the structure of TP4 in DPC micelles due to its well-dispersed NMR signals in the solution, although helical structures were also seen in solution containing SDS, sarkosyl or LPS by CD spectrum analysis. It contained a main α-helix and a short distorted helix with an angle of ~22.6^o^. Our mutation and function studies on TP4 showed the hydrophobic residues Ala12, Ala15, Ile16, Leu19, Ile20 in the main helix were critical for the α–helical structure which is prerequisite for hemolytic and bactericidal activities. The hydrophobic residues of the N-terminal hydrophobic core, Phe1, Ile2, Ile5, Ile6, Leu9, Phe10, as well as C-terminal cationic residues were also involved in the hemolytic activity and bactericidal activity (Table 2, Fig 8 and S2 Fig). As shown in Fig 10, the double minimum signals of CD spectrum of dN2, dN4, dC4 and A12I/A15I were stronger than that of wild type TP4. This results indicate that deletion of non-structural coil of N- and C-termini or increase of hydrophobicity in the main helix of TP4 may increase its helix content. However, the increase of hydrophobicity by deletions of non-structural coils at N- and C-termini reduced both bactericidal and hemolytic activities, while the increase of hydrophobicity in the main helix by A12I/A15I mutation reduced OprI-binding and bactericidal activity but increased hemolytic activity. These results indicate that the increase of hydrophobicity and helix content of an AMP are helpful for hemolytic activity, but not strictly correlated with its OprI-binding and antimicrobial activity. For example, the bulky isoleucine residues, A12I/A15I, on the hydrophobic face of TP4 main helix may not favor AMP assembly or OprI-binding, but are helpful for the integrity of helix and hemolysis. On contrast, deletion or substitution of terminal non-structural hydrophobic residues is detrimental to both bactericidal and hemolytic activities.

It is interesting to investigate the roles of His3 and His4 on the antimicrobial and hemolytic activities of TP4 because further deletion of N-terminal two residues (dN4) was able to restore the lost bactericidal activity of dN2 mutant. Among the TP4 variants tested so far, only H3A/H4A mutant lost fungicidal activity but it retained bactericidal activity as well as hemolytic activity. It is suggested that the bactericidal activity of TP4 is attributed to the adherence of its N-terminal hydrophobic core to bacterial membrane/target protein, but the interaction may be hindered by exposed hydrophilic His3/His4 residues at N-terminus of dN2 mutant, and it could be restored by further deletion of two hydrophilic residues (dN4) having hydrophobic Ile5/Ile6 residues exposed. Of note is the different susceptibility of Gram-negative, -positive bacteria and fungi to various TP4-derived peptides (Table 2 and S2 Fig). For example, dC4 is bactericidal to Gram-positive *S*. *aureus* and MRSA but not to Gram-negative *P*. *aeruginosa*. Thus it is suggested that the targets of TP4 and even its responsible residue for antimicrobial activity varied with microbes it attacks. The study provides valuable insight for engineering AMP with high antimicrobial activity but low hemolytic or cytotoxic activity.

**Fig 11:**
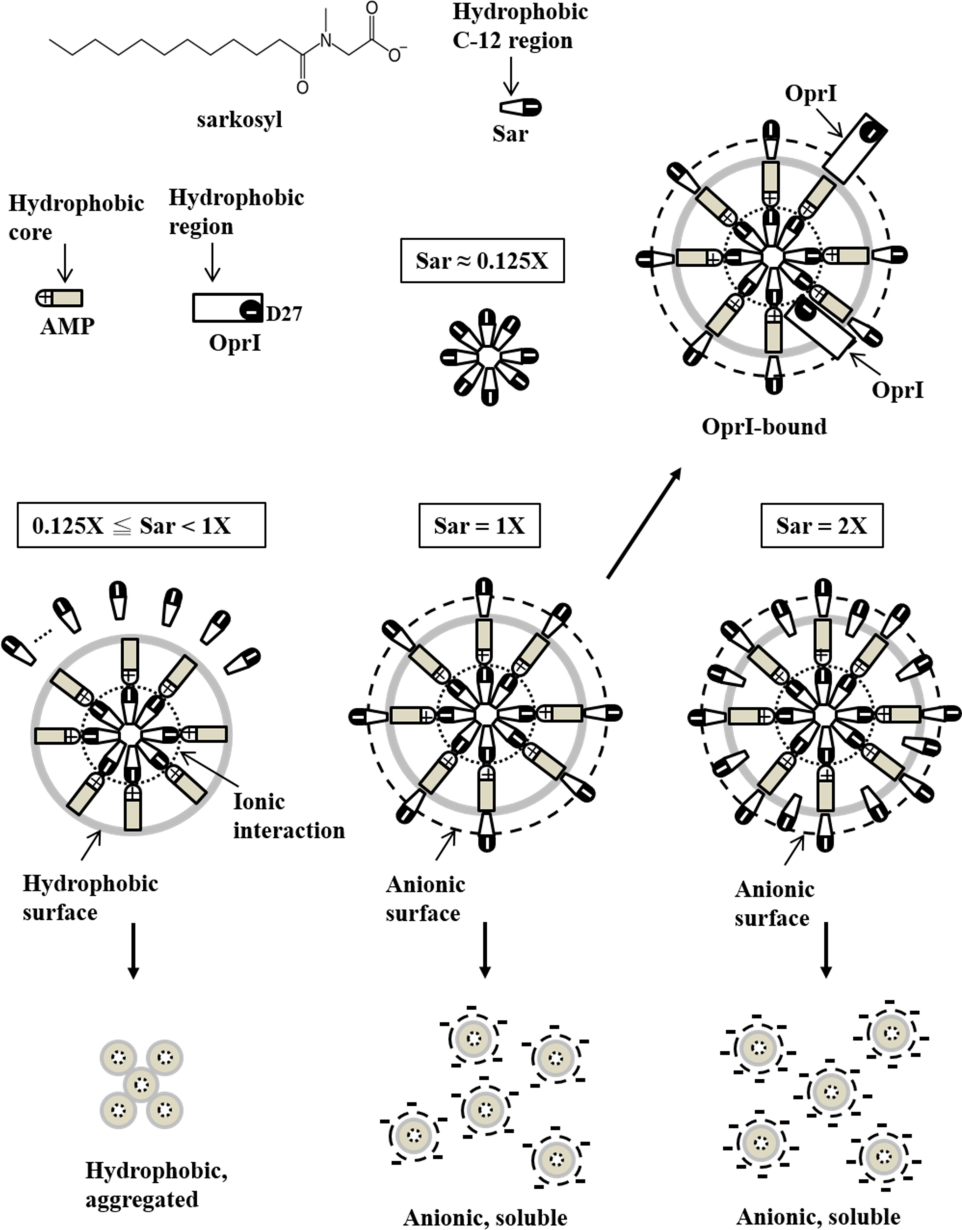
Proposed model of TP4 structure induced by sarkosyl. The sarkosyl molecules form micelle having hydrophobic C-12 tail in the center and anionic group outside at 0.125x Sar. The TP4 molecules are adhered to the anionic surface of micelles through ionic interaction leaving hydrophobic surface exposed to aqueous solution at 0.125-1x Sar. Under this environment, the surface of hydrophobic TP4 was not fully cover by sarkosyl, thus they are insoluble and aggregated. Once the sarkosyl concentration increases to 2.6 mM (1x Sar), the TP4 assemblies are fully coated by sarkosyl through hydrophobic interaction and lined up in a concentric vesicle and become soluble again due to charge repulsion on surface. The binding of OprI to TP4 in 1x Sar is suggested to mediate through head to head or side by side hydrophobic interactions. By the way, ionic interaction may also involve in the binding between cationic residues of TP4 and the anionic residue of OprI, Asp27. However, the hydrophobic interaction may be inhibited by higher concentration of sarkosyl (2x Sar).

Our study demonstrated that the interaction between TP4 and its receptor OprI was dramatically enhanced by the two agents, sarkosyl and lipopolysaccharide, due to structure conversion. The conversion from random-coil to α-helix by sarkosyl was reversible depending on the concentration of sarkosyl employed. Interestingly, the binding of TP4 to OprI was not enhanced by other anionic surfactants like SDS and cationic surfactant DPC used for this NMR study (Fig 5) even though they have been shown to induce helix formation (Fig 3). Sarkosyl is composed of a 12-carbon hydrophobic tail and an anionic carboxylate head-group tethered by an amide group (Fig 11), while SDS is composed of a 12-carbon hydrophobic tail attached to a sulfate group only. The induction of helical structure of TP4 may be driven by the hydrophobic force of the hydrophobic tail of sarkosyl on the hydrophobic residues of TP4, as well as the electrostatic interaction of anionic group of sarkosyl on cationic residues of TP4. The surfactant DPC employed in this report for NMR study and PRE experiments is also composed of a 12-carbon hydrophobic tail attached to a phosphatidylcholine group. The concentration of DPC employed in this study (100 mM for NMR, 15 mM for CD) was much higher than its CMC value (1.5 mM in water), in which TP4 was unable to bind OprI. The binding of TP4 to OprI was not enhanced by SDS or DPC at the respective concentrations as that of sarkosyl employed in this study. The defective effect of SDS and DPC on the binding of TP4 to OprI may due to the presence of sulfate and phosphatidylcholine group, instead of carboxylate group tethered by amide group at the hydrophilic head.

With respect to the characteristics of other surfactants, it is known that both anionic surfactants (SDS, sarkosyl) and non-ionic surfactants (Triton X-100, Tween 20) can be employed to solubilize or extract proteins from inclusion bodies or membrane fractions of tissues at higher concentrations. However, they are also used to protect or stabilize proteins from urea- and thermal induced denaturation. Currently, some anionic bio-surfactants such as sarkosyl with an amide group are frequently used in the industry for making small-particle emulsions for cosmetics. Sarkosyl could self-assemble into oligomers in the form of monomers, micelles, inter-digitations or fully developed bilayers depending on the concentration of counter ions in the surfactant solution. The critical micelle concentration (CMC value) of sarkosyl in 20 mM phosphate, pH 7.9 is 6.2 mM which is less than that obtained in pure water with 13 mM due to increased presence of the counter ion (Na^+^). As the concentration of sarkosyl reaches 5 mM in 20 mM phosphate, pH 7.9, it starts to destroy the α-helical structure of bovine serum albumin and completely disrupt the protein structure at 12 mM [30,31]. Here, the optimal concentration of sarkosyl to enhance OprI-binding is 2.6 mM in the buffer containing 10 mM sodium phosphate, pH 7.4 and 0.1M NaCl, which may be closed to the CMC values (lower than the reported values, 6 mM and 13 mM for phosphate buffer and pure water, respectively).

Due to the low solubility of TP4 (less than 80 μM) in 1x Sar (2.6 mM sarkosyl), no well-dispersed NMR signals were obtained at higher concentration such as 1.5 mM TP4 in 2.6 mM sarkosyl as that obtained in 100 mM DPC. Several surfactant-like amphipathic peptides have been found to self-assemble into nanotubes or nanovesicles. The transitions of these two conformations, even in monomer, are reversible depending on pH value, concentration of salt or peptide in the environment [32–34]. Based on our CD spectrum, ANS blue shift, precipitation and OprI-binding experiments, the conformation of 60 μM TP4 in different sarkosyl concentrations are suggested to exist in the following forms as shown in Fig 11. First, the sarkosyl molecules start to form micelle with hydrophobic C-12 tail in the center and anionic group outside at 0.125x Sar. Second, the random-coiled AMPs are induced to form amphipathic structure and adhered to the anionic surface of existing micelles through electrostatic interaction at 0.125x-1 x Sar, thus leaving hydrophobic face exposed to the aqueous solution. Due to the concentration of sarkosyl is not sufficient to cover the whole surface of hydrophobic AMP assembly, these AMPs are insoluble, aggregated and precipitated in aqueous solution although they may exist in an α–helical structure. Third, the AMPs are fully coated and solubilized by sarkosyl through hydrophobic interaction once the sarkosyl concentration increases to 2.6 mM (1x Sar). Thus the resulting sarkosyl-AMP-sarkosyl assemblies line up in a concentric vesicle and become soluble again due to charge repulsion on surface. The binding of OprI to TP4 in 1x Sar is suggested to mediate through head to head or side by side hydrophobic interactions. Ionic interaction may also involve in the binding between cationic residue of TP4 and the anionic residue of OprI, Asp27 [17]. However, the hydrophobic interaction between OprI and TP4 may be inhibited by higher concentrations of sarkosyl, 2x Sar (5.2 mM) or more, although the TP4 still remains in an α–helical structure. With regards to the helical structure of TP4 driven by LPS, it had increased hydrophobicity (analyzed by ANS blue shift and precipitation test Fig 4B and 4D) in phosphate buffer. It is suggested that the amphipathic structure of TP4 is induced by the hydrophobic lipid A region of LPS and bound to the anionic phosphate group of LPS by electrostatic interaction. Therefore, the hydrophobic lipid A region of LPS as well as hydrophobic face of TP4 are exposed and precipitated in the aqueous solution.

Depolarization or disruption of the cytoplasmic membrane potential renders the bound fluorescent dye DiSC3(5) to be released into medium leading to increased intensity of fluorescence [35]. Similar to most AMPs, TP4 caused depolarization of membrane potential once the bacterial membrane was permeabilized or disrupted beyond lethal dose [10]. However, the membrane potential of *P*. *aeruginosa* was hyperpolarized by TP4 at a sub-lethal dose which also occurred in *P*. *aeruginosa* treated by the bactericide granulysin [11] (Fig 1B and 1C). Although depolarization of membrane potential is considered to be an initial event in membrane injury, hyperpolarization has also been reported to be an adaptive behavior before cell death pathways are triggered [36–38]. Therefore, it is implicated that the treatment of AMPs at a sub-lethal dose may enable the bacteria to trigger an adaption pathway and develop drug-resistant mechanisms.

In conclusion, the structure of TP4 contains a main α-helix, a distorted helix, an N-terminal hydrophobic core and a C-terminal cationic patch. TP4 was driven into a helix by the membrane-like surfactant sarkosyl or LPS before binding to target receptor. The hydrophobic residues residing on the main helix are critical for its amphipathic structure as well as its hemolytic and bactericidal activities.

## Supporting information

S1 Fig The effects of paramagnetic probes on TP4 in DPC micelles were investigated by using Mn^2+^ ions. All spin labels are at the concentration of 1.15 mM.
(TIF)

S2 Fig Inhibition zone assay of TP4-derived peptides. Five ml of low melting agar (1%) mixed with 100 μl of overnight-cultured microbes was spread on 1% regular agar plate. Two μl of diluted TP4-derived peptides (0.5 and 2 μg/μl, inside and outside row, respectively) were dotted on the top layer of microbe-containing agar plate and incubated overnight at 30/37 ^o^C for the counting of colonies. MRSA represents methicillin-resistant *Staphylococcus aureus*. ddH2O was used as a negative control.
(TIF)

S3 Fig Binding of TP4-derived peptides to bacteria. Overnight cultures of Gram-negative *P*. *aeruginosa* (A) and Gram-positive *S*. *aureus* (B) (10^7^ cfu) were incubated with TP4-derived peptides (4 μg each) in 50 μl at 37°C for 30 min, then spun at 3,300 x *g* for 10 min followed by SDS-PAGE and Coomassie blue staining. C, peptide control; P, pellet; P.a, *P*. *aeruginosa*; S.a, *S*. *aureus*.
(TIF)

S4 Fig ANS emission spectra of TP4-derived peptides. TP4-derived peptides (4 μg each) were dissolved in 200 μl of PC buffer (A) or 1x sarkosyl solution (B). ANS was added stepwise to the final concentrations as indicated (lines 1 to 6 at 0, 10, 20, 30, 40 and 50 μM, respectively). Arrows indicate the emission maximum at 470 nm or 520 nm of bound- and free-form ANS, respectively.
(TIF)
