# Supplementary figures and images for: Hydrophobic residues are critical for the helix-forming, hemolytic and bactericidal activities of amphipathic antimicrobial peptide TP4
Source: PLoS One. 2017 Oct 17;12(10):e0186442. doi: 10.1371/journal.pone.0186442 (PMC5645128; doi:10.1371/journal.pone.0186442)

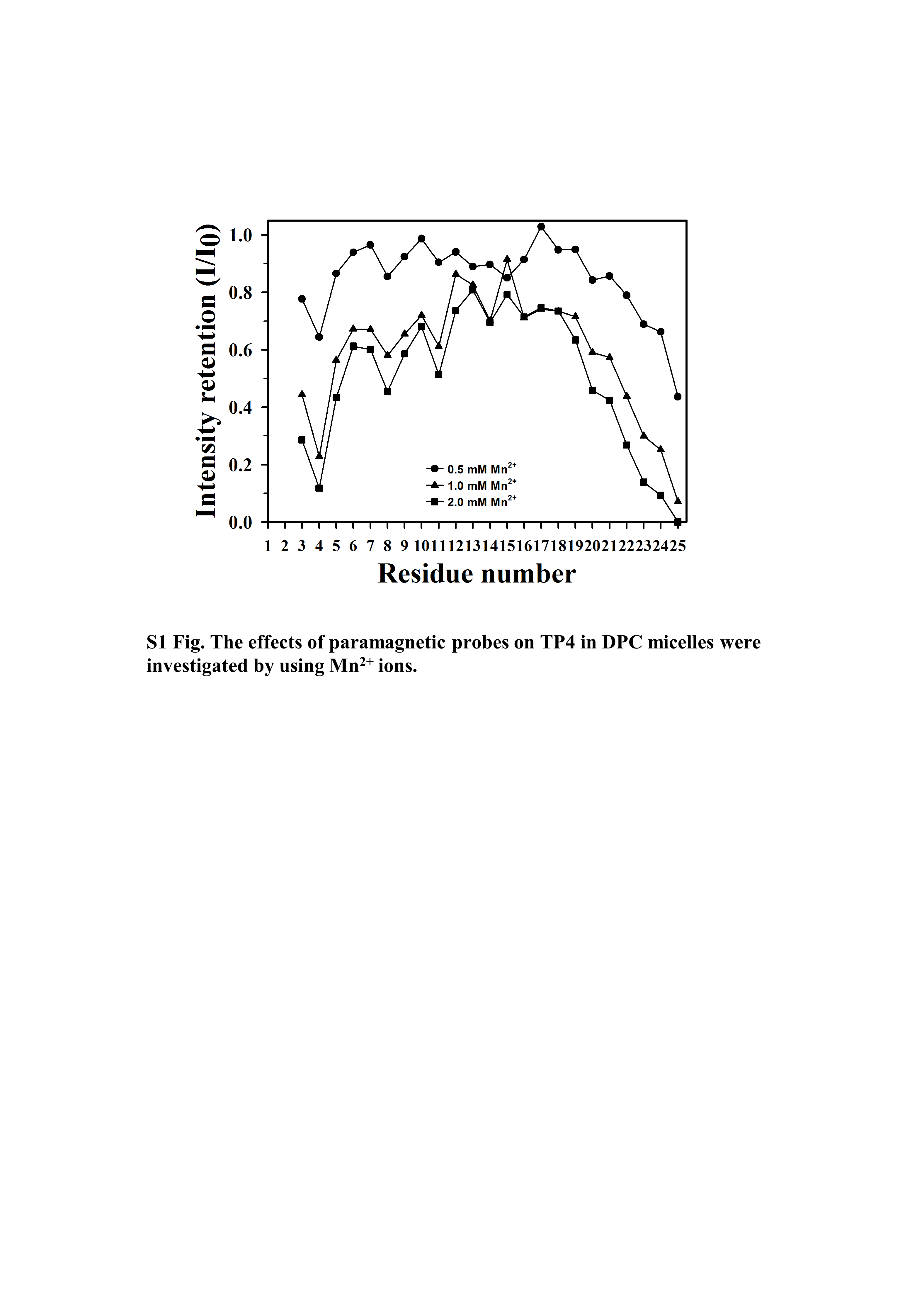

Supplement: S1 Fig — All spin labels are at the concentration of 1.15 mM. (TIF) [file pone.0186442.s001.tif]

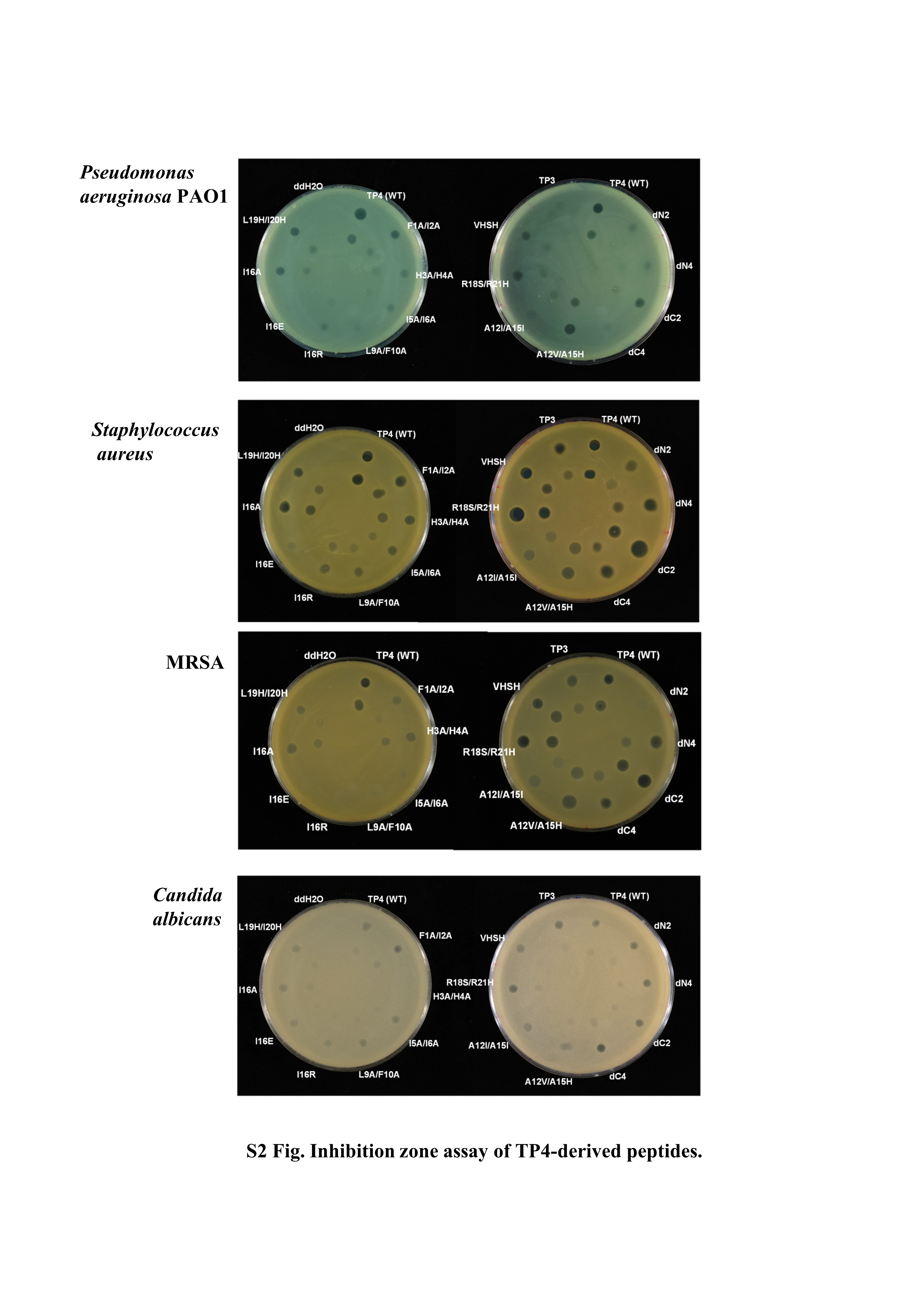

Supplement: S2 Fig — Five ml of low melting agar (1%) mixed with 100 μl of overnight-cultured microbes was spread on 1% regular agar plate. Two μl of diluted TP4-derived peptides (0.5 and 2 μg/μl, inside and outside row, respectively) were dotted on the top layer of microbe-containing agar plate and incubated overnight at 30/37 oC for the counting of colonies. MRSA represents methicillin-resistant Staphylococcus aureus. ddH2O was used as a negative control. (TIF) [file pone.0186442.s002.tif]

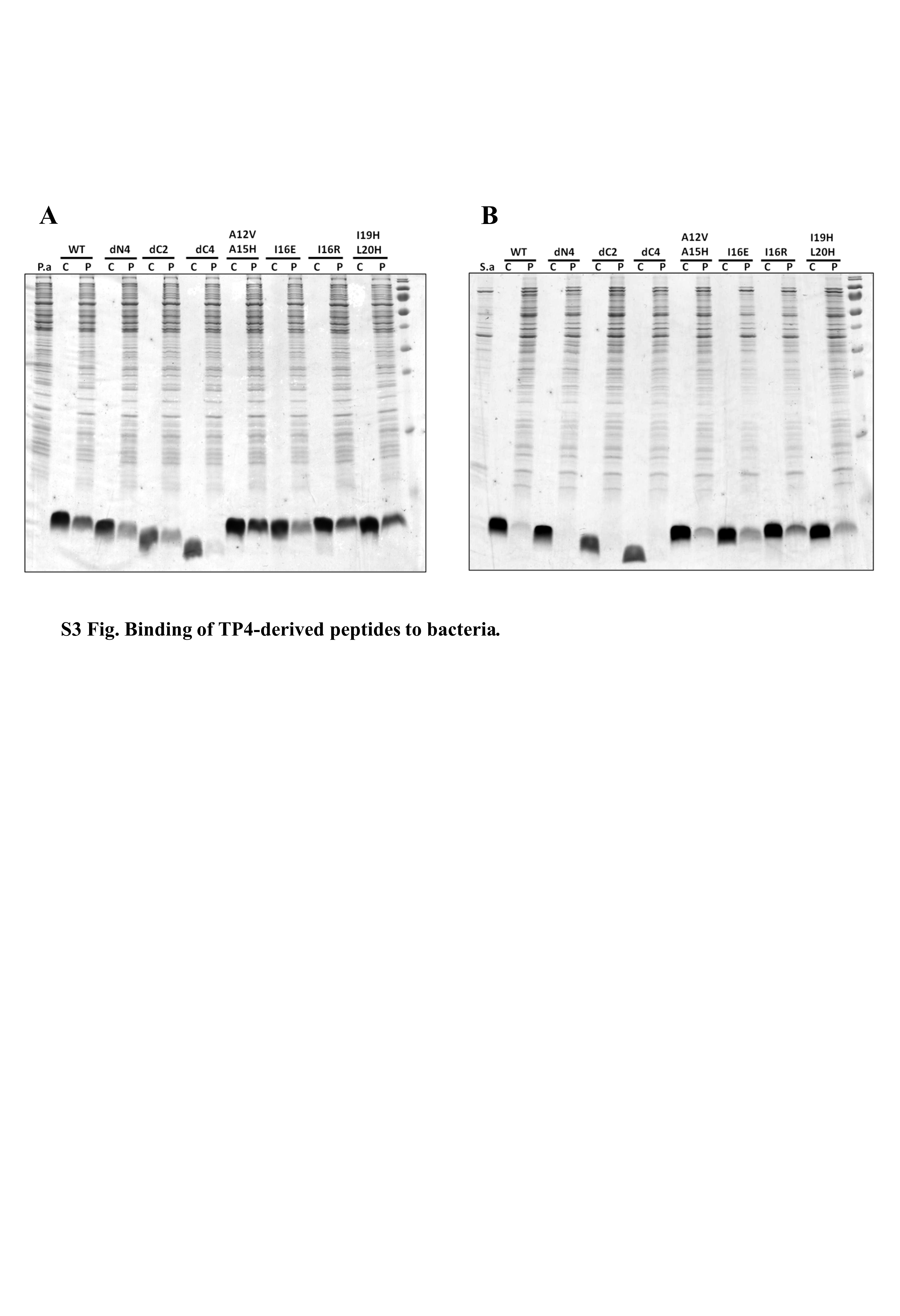

Supplement: S3 Fig — Overnight cultures of Gram-negative P. aeruginosa (A) and Gram-positive S. aureus (B) (107 cfu) were incubated with TP4-derived peptides (4 μg each) in 50 μl at 37°C for 30 min, then spun at 3,300 x g for 10 min followed by SDS-PAGE and Coomassie blue staining. C, peptide control; P, pellet; P.a, P. aeruginosa; S.a, S. aureus. (TIF) [file pone.0186442.s003.tif]

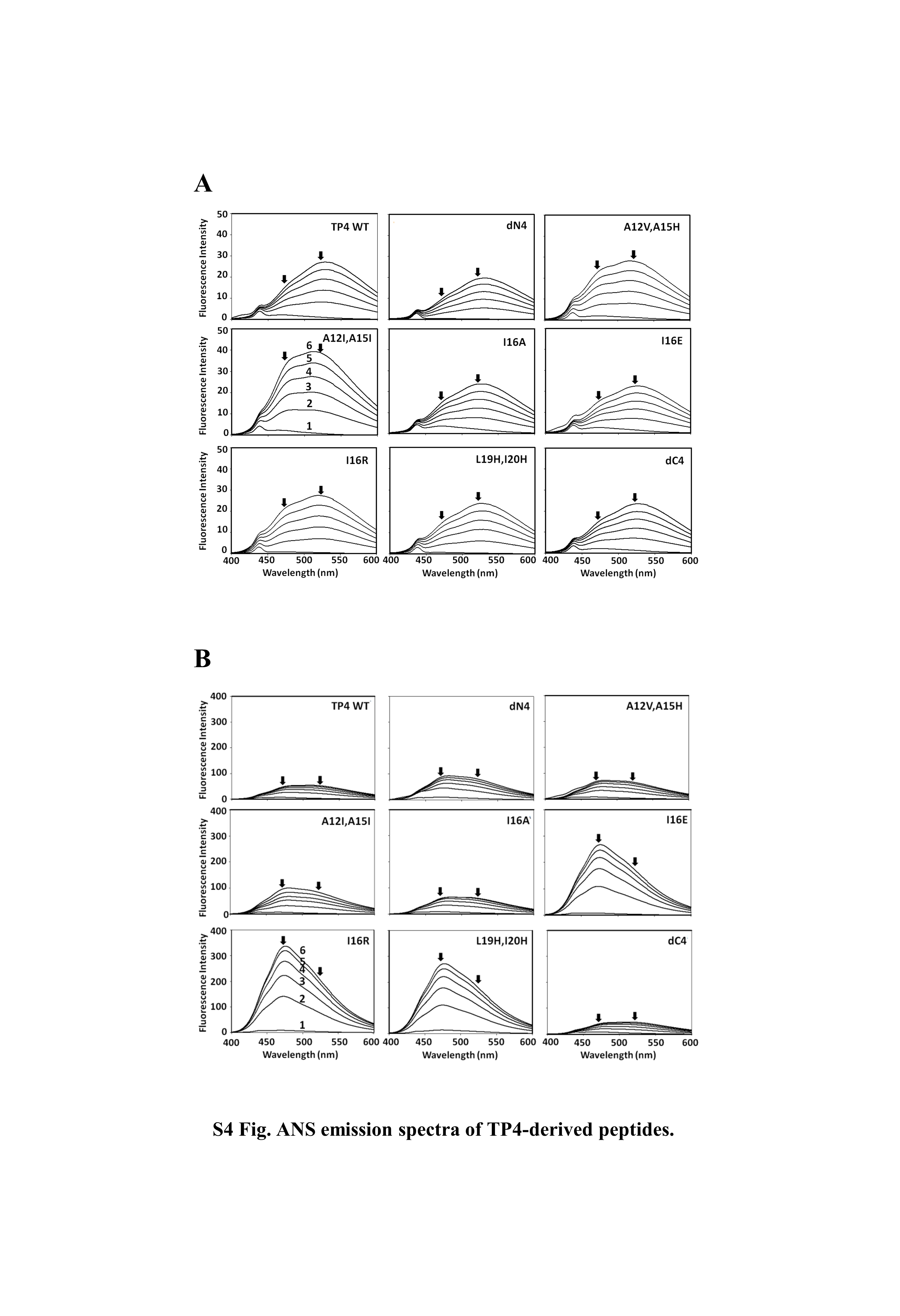

Supplement: S4 Fig — TP4-derived peptides (4 μg each) were dissolved in 200 μl of PC buffer (A) or 1x sarkosyl solution (B). ANS was added stepwise to the final concentrations as indicated (lines 1 to 6 at 0, 10, 20, 30, 40 and 50 μM, respectively). Arrows indicate the emission maximum at 470 nm or 520 nm of bound- and free-form ANS, respectively. (TIF) [file pone.0186442.s004.tif]
